# Isoprene emission by poplar is not important for the feeding behaviour of poplar leaf beetles

**DOI:** 10.1186/s12870-015-0542-1

**Published:** 2015-06-30

**Authors:** Anna Müller, Moritz Kaling, Patrick Faubert, Gerrit Gort, Hans M Smid, Joop JA Van Loon, Marcel Dicke, Basem Kanawati, Philippe Schmitt-Kopplin, Andrea Polle, Jörg-Peter Schnitzler, Maaria Rosenkranz

**Affiliations:** Büsgen Institute, Forest Botany and Tree Physiology, University of Göttingen, Büsgenweg 2, 37077 Göttingen, Germany; Research Unit Environmental Simulation, Institute of Biochemical Plant Pathology, Helmholtz Zentrum München - German Research Center for Environmental Health (GmbH), Ingolstädter Landstraße 1, 85764 Neuherberg, Germany; Research Unit Analytical BioGeoChemistry, Helmholtz Zentrum München, D-85764 Neuherberg, Germany; Département des Sciences Fondamentales, Chaire en éco-conseil, Université du Québec à Chicoutimi, 555, boul. de l’Université, Chicoutimi, Qc G7H 2B1 Canada; Mathematical and Statistical Methods Group, Wageningen University, P.O. Box 100, 6700 AC Wageningen, Netherlands; Laboratory of Entomology, Wageningen University, P.O. Box 8031, NL-6700 EH Wageningen, Netherlands

**Keywords:** *Chrysomela populi*, Volatile organic compounds, VOC, Isoprene, Isoprenoids, Terpene, Electroantennography, Beetle behaviour, *Populus* x *canescens*, Metabolomics

## Abstract

**Background:**

*Chrysomela populi* (poplar leaf beetle) is a common herbivore in poplar plantations whose infestation causes major economic losses. Because plant volatiles act as infochemicals, we tested whether isoprene, the main volatile organic compound (VOC) produced by poplars (*Populus* x *canescens*), affects the performance of *C. populi* employing isoprene emitting (IE) and transgenic isoprene non-emitting (NE) plants. Our hypothesis was that isoprene is sensed and affects beetle orientation or that the lack of isoprene affects plant VOC profiles and metabolome with consequences for *C. populi* feeding.

**Results:**

Electroantennographic analysis revealed that *C. populi* can detect higher terpenes, but not isoprene. In accordance to the inability to detect isoprene, *C. populi* showed no clear preference for IE or NE poplar genotypes in the choice experiments, however, the beetles consumed a little bit less leaf mass and laid fewer eggs on NE poplar trees in field experiments. Slight differences in the profiles of volatile terpenoids between IE and NE genotypes were detected by gas chromatography - mass spectrometry. Non-targeted metabolomics analysis by Fourier Transform Ion Cyclotron Resonance Mass Spectrometer revealed genotype-, time- and herbivore feeding-dependent metabolic changes both in the infested and adjacent undamaged leaves under field conditions.

**Conclusions:**

We show for the first time that *C. populi* is unable to sense isoprene. The detected minor differences in insect feeding in choice experiments and field bioassays may be related to the revealed changes in leaf volatile emission and metabolite composition between the IE and NE poplars. Overall our results indicate that lacking isoprene emission is of minor importance for *C. populi* herbivory under natural conditions, and that the lack of isoprene is not expected to change the economic losses in poplar plantations caused by *C. populi* infestation.

**Electronic supplementary material:**

The online version of this article (doi:10.1186/s12870-015-0542-1) contains supplementary material, which is available to authorized users.

## Background

Isoprene is a volatile organic compound (VOC) emitted in large quantities by fast growing tree species, such as poplar, willow and oil palm [1]. Isoprene affects the chemistry of the troposphere by contributing to ozone production, secondary organic aerosol (SOA) formation and by enhancing the lifetime of methane [2–4]. Considering the growing interest in biomass production by poplar plantations, genetically modified, isoprene non-emitting (NE) poplar trees could help to prevent atmospheric pollution and climate change [5, 6].

VOCs play important roles in the interaction between plants and herbivores. Induced VOCs are released from plants during and following abiotic or biotic stresses, such as high temperature episodes or insect feeding [5, 7, 8]. Plants, insects and microbes can interact with community members by volatile compounds [9–12]. Insects can recognise individual molecules with their olfactory receptor neurons (ORN) [13]; however, a correct mix of volatiles is typically necessary to react to an odour blend [14]. Common insect-induced plant VOCs are green leaf volatiles (GLVs) and mono- and sesquiterpenes [7].

A previous study demonstrated that isoprene emitted by transgenic Arabidopsis interferes with the attraction of *Diadegma semiclausum* (Hymenoptera, Ichneumonidae), a parasitic wasp searching for its herbivorous host [15]. Moreover, it was shown that isoprene-emitting transgenic tobacco plants are less attractive for tobacco hornworm (*Manduca sexta* Lepidoptera, Sphingidae) than unmodified tobacco plants [16]. A further indication that isoprene may act as a repellent comes from a study with transgenic poplars showing that Brassy willow beetles (*Phratora vitellinae*,; Coleoptera, Chrysomelidae) were more abundant on NE poplars than on isoprene emitting (IE) plants under field conditions [6]. Whether the preference of *P. vitellinae* was due to the altered isoprene emission capacity or due to other yet unrevealed biochemical factors, remained unexplored in this study [6].

The poplar leaf beetle (*Chrysomela populi*,; Coleoptera, Chrysomelidae) as well as *P. vitellinae* are common leaf-feeding beetles highly abundant in poplar plantations. They are particularly found on young trees where they can cause high economic losses [17–19]. Both larvae and adults of the species feed on the leaf material, particularly on young leaves. Commonly, members of the Chrysomelidae are highly specialised herbivorous beetles [18]. *C. populi* and *P. vitellinae* are specialists using salicyl glucosides from the host plant to build their own defence [20]. In previous olfactometer studies, it was shown that *C. populi* uses monoterpenes and sesquiterpenes to search for young but not fully mature poplar leaves [21] that are rich in salicylic glucosides [22].

In the present study, biochemically and transcriptionally well-characterised NE and IE poplar genotypes [23, 24] are used aiming to clarify the potential bioactive function of isoprene in a widespread, common plant-herbivore interaction. We investigated whether (i) *C. populi* is able to detect isoprene and other volatile terpenoids typically emitted by poplar leaves following herbivory, (ii) the suppression of isoprene emission in NE poplars affects the plant VOC profile, metabolome or biomass, (iii) the absence of isoprene affects the behaviour and fitness of *C. populi* on NE poplars. To study this, we conducted electroantennography (EAG), feeding choice and growth experiments under greenhouse conditions, analysed the VOC emission profile of infested and non-infested genotypes, tested the feeding choices of leaf beetles and analysed plant metabolomic adjustments and biomass of the IE and NE poplar trees in caged field exposure systems.

We report that *C. populi* and *P. vitaellinae* are not able to detect isoprene. Despite of that *C. populi* slightly preferred IE over transgenic NE poplars, which might be due to alterations we reveal in leaf volatile emission and metabolite composition between the poplar genotypes. We report, in addition, time- and herbivore feeding-dependent metabolic changes both in the infested and adjacent undamaged poplar leaves under field conditions

## Materials and methods

### Plant and insect material

Transgenic non-isoprene emitting (NE) poplars (genotypes RA1, RA2 and RA22,) and isoprene emitting (IE) wild type and transgenic, vector control Grey poplar trees (*Populus x canescens*; Aiton (Sm) syn. *Populus tremula L. x P. alba L.*) were used. In the NE genotypes, the isoprene synthase expression was silenced by the RNA interference (RNAi) technique [5, 6, 23, 25]. As transgenic, isoprene emitting, vector controls β-glucuronidase (GUS)/green fluorescent (GFP) (GUS and GFP genes in one vector) under *Populus* x *canescens* isoprene synthase (*Pc*ISPS) promoter expressing poplar trees were used [26]. For more details on the NE genotypes, please see Behnke et al. [5] and for more details on the IE genotypes see Cinege et al. [26] and Way et al. [27].The genotypes used in each experiment are presented in the Additional file 1: Table S1. When no differences in VOC emissions and herbivore behaviour among the IE or the NE genotypes were found, the results of IE or NE genotype in each experiment were pooled. Plant cultivation and growth conditions before the experiments were as previously described for greenhouse [5, 26] and field conditions [28]. The trees were used in experiments when they had reached a height of approximately 60–80 cm and 20 leaves.

*Chrysomela populi* were collected in poplar plantations in Southern Germany near Freising, Scheyern and Sigmaringen. For larval bioassays, eggs were collected and larvae allowed hatching on its host leaf whose petiole was placed into 1 % agar solution to avoid drying in a greenhouse. For the bioassays, either 1^st^ instar larvae or 3^rd^ instar larvae were used. The 1^st^ instar larvae were allowed to eat the remains of the egg before the initiation of the experiments. The 3^rd^ instar larvae had previous experience on IE or NE plants before the beginning of the experiment to investigate if an effect of feeding experience occurred. The bioassays with adult beetles were performed with overwintered insects except for EAG and choice-studies, in which the 1^st^ new generation of the summer was used, and olfactometer experiments, in which the 2^nd^ generation of the summer was used. For rearing conditions please see Additional file 1: Table S1. In each experiment the beetles were randomly assigned to different plants in the experimental design to minimize possible effects of previous experience, beetle age or sex, similar as described in Hjälten *et al*. [29].

*Phratora vitellinae* L. (Brassy willow beetle) adults for additional EAG studies were collected in the field on Grey poplars at Göttingen University.

### Electroantennography

Electroantennography (EAG) recordings were performed as described in [30]. The responses of *C. populi* and *P. vitellinae* individuals to isoprene, α-pinene, β-pinene, ocimene, linalool, β-caryophyllene and methyl salicylate were recorded. The compounds were applied as described in Loivamäki et al. [15] with following changes: Stimulus puffs (0.5 sec, 120 ml min^−1^) were injected into air of 600 ml min^−1^ running over the antennal preparation, consisting of one, from the head separated, antenna. The green leaf odour (*Z*)-3-hexen-1-yl acetate (≥98 % purity, Sigma–Aldrich; 10 % solution in hexadecane) was used as a standard odour. The standard odour was applied in the beginning and end of one series that involved five different volatile compounds in the three different concentrations in ascending order and the control stimulations. Control stimulations were performed with 10 μL of hexadecane. Both standard and control odours were applied before and after the series of stimulations of each compound dilution.

### Volatile collection and analysis by gas chromatography–mass spectrometry (GC-MS)

For VOC collection by push-pull technique [31, 32], two plants of each genotype and herbivore treatment (uninfested trees and 10 *C. populi* adult 24 h freely infested trees ) were placed in parallel in two glass cuvettes (volume of 60 L; air temp. 23 °C; approx. 200 μmol photons m^−2^ s^−1^, schema of the cuvette in Additional file 2: Fig. S1). To avoid VOC emissions and evaporation from soil, the pot and soil were covered by aluminium foil. After acclimation, the system was purged for 30 min with 2000 ml min^−1^ synthetic air mixed with 370 ppm CO_2_. Headspace volatiles from non-infested and infested IE and NE genotypes were collected for a period of 2 h between 9:30 AM and 11:30 AM (*n* = 6). Air was pumped out of the cuvette with 100 ml min^−1^ by passing through a tube filled with polydimethylsiloxane (PDMS) (Gerstel GmbH & Co. KG, Mülheim an der Ruhr, Germany) and 50 mg of Carbopack B (mesh 60/80; GraceAlltech, Düsseldorf, Germany). The overall leaf areas of each tree were determined immediately after the experiments using a portable Area Meter (LI-COR, Walz, Effeltrich, Germany).

The VOC samples were analysed with a thermo-desorption unit (Gerstel) coupled to a gas chromatograph-mass spectrometer (GC-MS; GC model: 7890A; MS model: 5975C; Agilent Technologies, Santa Clara, CA, USA). VOCs were desorbed from 30 °C to 270 °C at a rate of 400 °C min^−1^, followed by a holding time of 3 minutes. The compounds were refocused on Tenax (cryo-cooling technique) at −50 °C and desorbed to 250 °C at a rate of 10 °C s^−1^, followed by a holding time of 5 minutes. Compounds were separated as described in Müller *et al*., [33].

The VOCs were extracted from the data as described in Müller *et al*. [33]. The presence of previously reported VOCs of poplar [21, 25, 34, 35] were also proved in the emission profile. The representative m/z and retention indices of the remaining VOCs were calculated according to van Den Dool and Kratz [36, Additional file 3: Table S2].

The TIC of each VOC in the final dataset was recalculated as described in Müller *et al*., [33] and normalised to overall leaf area. Individual compounds were grouped in monoterpenes, sesquiterpenes and other VOCs (OVOCs) [Additional file 4: Table S3]. Quantification of the compound concentrations was conducted using the TIC of external standards: isoprene and α-pinene for non-oxygenated monoterpenes, linalool for oxygenated monoterpenes, (*E*)-caryophyllene for non-oxygenated sesquiterpenes, nerolidol for oxygenated sesquiterpenes and toluene for OVOCs.

### Olfactometer Bioassays

The behavioural response of adult *C. populi* to plant volatiles was investigated in a Y-tube olfactometer [37, Additional file 2: Fig. S1] under constant conditions (22 °C). The studies were conducted immediately after VOC collection from the poplar genotypes with the same set up and a flow of 500 ml min^−1^ from IE trees to one of the side arms and 500 ml min^−1^ from NE trees into the other side arm of the Y-tube. One beetle at a time was introduced into the downwind part of the olfactometer using a glass vial, and the beetle’s behaviour was observed for a maximum of 10 min. A choice was recorded when the beetles stayed at a maximum of 2.5 cm distance from an end of one of the Y-tube side arms for 15 seconds. Beetles that did not make a choice within 10 min were discarded from the statistical analysis. Twenty beetles were tested per day and plant set-up. Altogether we tested 122 beetles. To correct for unforeseen asymmetry in the set-up, the position of the odour sources was switched after every five beetles tested.

### *C. populi* bioassays under greenhouse conditions

The choice-studies on leaves were performed with 3^rd^ instar larvae and with adult *C. populi* beetles that had previous experience on either NE or IE plants (Additional file 1: Table S1). The studies were performed in plastic boxes (16 cm × 11 cm × 32 cm (depth × height × width)) with holes on the top. In the box, individual leaves of IE and NE poplars were placed opposite to each other. To avoid leaf desiccation, petioles were placed in water in a 2 ml Eppendorf tube (Sarstedt, Nümbrecht, Germany). Moisturised tissue was placed at both ends of the box to provide sufficient humidity in the box. A thin cloth stretched over the top of the box prevented the insects from escaping and to distribute the sunlight evenly. To further create homogeneous environmental conditions, the boxes were placed in bigger, 32 cm × 39 cm × 60 cm (depth × height × width), light impermeable open top boxes. These open top boxes were placed in the climate controlled (19 ± 1 °C; air humidity 50 %) acrylic glass cuvettes (size: 80 cm × 110 cm × 110 cm (depth × height × width, Additional file 5: Fig S2) in a greenhouse under natural day light. At the start of recording, one insect was placed in the middle of the box. The first choice of the larvae was recorded, and the leaf feeding preference of larvae and adults was determined after 48 h by analysing the leaf area from the photographs by SigmaScan Pro5 (Systat Software, California, USA). The studies were conducted in the years 2012 and 2013 for larvae and adults, respectively.

To analyse the feeding choices of adult *C. populi* on whole poplar trees, two IE and two NE plants were placed in acrylic glass cuvettes (Additional file 5: Fig S2) at 19 ± 1 °C with a rel. air humidity of 50 % in a greenhouse under natural daylight. Only the shoots were inside the cuvettes and were separated by acrylic glass from the belowground parts (= pot with the roots in the soil) (Additional file 5: Fig S2). The bioassays were started by releasing 19 adult beetles and allowing them to feed freely over a 48-h period. The location of the beetles was recorded at hours 0, 0.5, 2, 6, 24, 30 and 48 after the beginning of the experiment. At the end of the experiment, the beetles were removed, and all individual leaves were photographed. The leaf area consumed was determined from the photographs as described above. Twenty individual experiments were performed during six weeks in May and June, 2012.

The growth and feeding preference of *C. populi* larvae were analysed on whole poplar trees. Ten 1^st^ instar larvae were positioned in the middle part, i.e., on the 7^th^ and 8^th^ leaves from the apex, of each tree. Each cuvette contained two trees of the same genotype and twenty larvae. The development was monitored for 10 days by weighing (Type r180 D, Sartorius GmbH, Göttingen, Germany) the larvae in the beginning and every 3^rd^ day during the experiments. After 10 days, the infested leaf area was recorded as described above, and the data of one cuvette was pooled. Eight individual experiments for each genotype were performed in June and July, 2011.

In all the greenhouse bioassays the roof of the greenhouse was covered by light non-permeable curtains avoiding direct sunlight. The duration of all the studies were chosen either for 24 or 48 hours that all possible sun possibilities (night, sunrise, high light and sundown) were experienced by the beetles. The studies were started around midday to avoid light variation during the first choices of the beetles. To correct for unforeseen symmetry the position of the IE and NE leaves/trees was switched in 50 % of the tests.

### *C. populi* bioassays under field conditions

Fourteen-day bioassays under field conditions were conducted in eight small cages (190 cm × 140 cm × 190 cm) covered with mesh screen (mesh size: 1.4 mm; thickness: 0.28 mm), which were located in Göttingen (Germany) in a caged area with permission to work with transgenic plants. Each small cage was equipped with four *P.* x *canescens* trees per genotype (NE and IE) three weeks before the start of beetle exposure (experimental design in Additional file 6: Table S4). The shoot height and diameter of stems were measured in the beginning and end of the 14-day experiment. At day zero (2 pm), 60 *C. populi* adult individuals were released in the middle of each of the four cages. We counted daily the number poplar leaves as well as number of herbivores and eggs on each leaf. The fed leaf area was visually quantified by using scale 1-to-5 in which:1: < 10 %, 2: 20-to-25 %, 3: 25-to-50 %, 4: 50-to-75 % and 5: > 75 % fed leaf area. Leaves were sampled and immediately frozen in liquid nitrogen on days 0, 2, 4 and 8 (at 2 pm). At each time point we sampled one infested (leaf number 5 from apex) and one adjacent undamaged leaf (one leaf between leaf number 10 and 15 from apex). For control we harvested leaves at the same positions from control trees without beetle exposure. Leaf samples of each genotype in one cage, with the upper and lower leaves separated, were pooled for FT-ICR/MS measurements. The leaves, stem, coarse roots and fine roots of all plants were weighed at harvest after two weeks of beetle exposure.

### Non-targeted metabolome analysis by FT-ICR/MS

For each sample, 20 mg of powdered leaf material was extracted two consecutive times with 1 ml −20 °C extraction solvent (methanol/isopropanol/water 1:1:1 [v/v/v]) in an ultrasonic bath for 15 min. Subsequently, the solution was centrifuged at 10,000 *g* for 10 min at 4 °C. The supernatant was removed and diluted with extraction solvent in a ratio of 1:25 (v/v). For non-targeted metabolome analysis, ultra-high-resolution mass spectra were acquired using a Fourier transform ion cyclotron resonance mass spectrometer (FT-ICR/MS, APEX Qe, Bruker, Bremen, Germany) equipped with a 12-Tesla superconducting magnet and an APOLLO II electrospray (ESI) source. Samples were introduced into the microelectrospray source (Agilent sprayer, Waldbronn, Germany) at a flow rate of 120 μl h^−1^ with a nebuliser gas pressure of 20 psi and a drying gas pressure of 15 psi at 180 °C using a Gilson autosampler (Sample changer 223, Gilson Inc., Middleton, USA). All measurements were performed in the negative ionisation mode over a mass range of m/z 100 – 1200. The spectra were acquired over a time domain transient of 2 Megawords with 488 accumulated scans for each sample.

For post-processing the measured mass spectra were aligned, internally calibrated and exported to peak height lists as ascii files at a signal to noise ratio of 4 and a relative threshold intensity of 1*10^−8^ % using the Data Analysis 4.1 software package (Bruker, Bremen, Germany). Peak lists were combined to a peak matrix with an error of 1 ppm using an in-house written tool [38]. Single mass events, isotopic peaks (^13^C, ^15^ N, ^34^S and ^18^O) and peaks detected in less than 50 % of the biological samples were removed from the matrix. The zero intensities present in the peak matrix were replaced with the FT-ICR/MS threshold intensity of 1*10^6^ counts. Next, the intensities were log^2^ transformed. For statistical analysis, poplar plants were grouped to their respective isoprene-emission capacity in IE (WT/GUS) and NE (RA1/RA22). To have a first overview on the FT-ICR/MS data, unsupervised principal component analysis (PCA) was performed using SIMCA-P 13.0 (Umetrics, Umeå, Sweden). To detect and identify masses (X-variables), which are important for the group separation, e.g., leaf age, herbivore treatment and isoprene emission capacity (Y-variables), supervised orthogonal partial least squares discriminant analysis (OPLS-DA) was applied. For each Y-variable, a separate OPLS-DA model was calculated and validated by seven cross-validation rounds. Masses with a variable influence of projection score (VIP) > 1 were extracted and further tested with a Wilcoxon-Mann–Whitney-U Test using Matlab R2011b (MathWorks, Ismaning, Germany). Masses with a VIP score >1 and a *P*-value <0.05 were considered as discriminant.

For metabolite identification, the mass list was uploaded to the MassTrix 3 server selecting negative ionisation mode, Kegg/HMDB/LipidMaps and MetaCyc as databases and *Populus trichocarpa* as organism [39, 40], as well as to the Metlin server [41]. The maximal error acceptance was 1 ppm.

### Statistical analyses

Mixed linear models were used to analyze the EAG data, because the experimental design resulted in repeated measurements on antennae and grouping of observations per day, leading to correlated responses. We followed the approach as described in Qiu *et al*. [42], applying the procedure MIXED of the SAS software program [43]. Mixed models contain a fixed part, capturing the experimental factors, as in ordinary linear models, and a random part, allowing for possibly complex correlations between observations. A detailed description of the model can be found in Additional file 7.

Binomial tests were performed to analyse the choices in the Y-tube olfactometer and choice-experiments. Wilcoxon signed-rank test was used to compare the infested leaf areas in the leaf choice-studies. A *t* test and a paired- sampled *t* test were used to compare the total infested leaf area in whole trees by larvae and adults, respectively. One-way ANOVA (analysis of variance) was used to analyse the larval weight at different time points. Instead of analyzing the original larval weight recordings, we analyzed ln(*y*) for normally distributed data. A Kruskal-Wallis and Mann Whitney *U* tests were applied to analyse VOC profiles between the genotypes and the herbivore-treated trees. A generalized linear model (GLM) was applied to analyse the infested leaf areas, eggs laid and beetle occurrence over time in the field experiment. For these statistical analyses, IBM SPSS Statistics version 21 (Armonk, NY, USA) was used.

## Results

### *C. populi* and *P. vitellinae* display olfactory sensitivity to higher isoprenoids but not to isoprene

Highly significant differences in EAG-responses were found between compounds, concentrations, and their combinations, and between the two species. Gender effects were weak: averaged over species, males tend to respond differently compared to females to (some) compounds (*P* = 0.02). All other interactions with gender are not significant (*P* > 0.05). The highly significant effects of order and order^2^ indicate the decrease of mean antenna sensitivity over time. The ANOVA table (Table 1) gives a compact overview on the importance of the different factors and their interactions.

**Table 1:** Results for F-tests on electroantennographic measurements

Effect	df1	df2	F value	P-value
species	1	78	1.46	0.23
gender	1	78	0.00	0.97
species × gender	1	78	1.90	0.17
comp	7	1620	273.2	<0.0001
conc	2	1620	144.5	<0.0001
comp × conc	12	1610	11.20	<0.0001
species × comp	7	1610	41.88	<0.0001
species × conc	2	1610	17.85	<0.0001
species × comp × conc	12	1610	5.50	<0.0001
gender × comp	7	1610	2.48	0.02
gender × conc	2	1610	0.35	0.71
gender × comp × conc	12	1610	0.72	0.72
species × gender × comp	7	1610	0.92	0.49
species × gender × conc	2	1610	0.81	0.44
species × gender × comp × conc	13	1610	0.45	0.94
order	1	87	132.4	<0.0001
order^2^	1	87	20.9	<0.0001

Results are shown for fixed effects of beetle species, gender, volatile compound (comp), concentration (conc), and their interactions;order of application and order^2^ are included as covariates in the mixed model

Absolute EAG response amplitude to the standard compound (*Z*)-3-hexenyl acetate (10 % concentration) was significantly different between species (*P* < 0.0001), but this species difference is not gender dependent (*P* = 0.23), nor did males and females respond differently (*P* = 0.32; Additional file 8: Table S5). For the response to the solvent hexadecane (at 100 %) no significant differences were found at all.

For both leaf beetle species and for all compounds, no significant interactions between factors dose and gender were found (*P* > 0.05). Thus, males and females responded with similar sensitivity to the compounds (Table 2).

**Table 2:** Results for statistical tests on dose-dependency of electroantenno-graphic responses

Species	Compound	dose–response	Δ dose–response	compound - solvent
		F + M	F/M	F + M
		(F-val/P-val)	(F-val / P-val)	(t-val / P-val)
		(df = 2,1610)	(df = 2,1610)	(df = 1610)
*C. populi*	isoprene	0.08 / 0.92	1.04 / 0.35	−3.90 / 0.0001
	linalool	86.87 / <.0001	0.80 / 0.45	20.61 / <0.0001
	methylsalicylate	19.07 / <.0001	1.13 / 0.32	17.54 / <0.0001
	ocimene	7.87 / 0.0004	1.65 / 0.19	5.06 / <0.0001
	α − pinene	183.76 / <.0001	0.29 / 0.75	28.85 / <0.0001
	β − caryophyllene	18.73 / <.0001	0.12 / 0.88	20.17 / <0.0001
	β − pinene	94.31 / <.0001	0.39 / 0.67	15.90 / <0.0001
*P. vitellinae*	isoprene	0.19 / 0.83	0.84 / 0.43	0.05 / 0.96
	linalool	18.66 / <.0001	0.01 / 0.99	12.90 / < 0.0001
	methylsalicylate	6.26 / 0.002	0.08 / 0.93	11.04 / 0.0001
	ocimene	0.66 / 0.52	0.64 / 0.53	6.05 / <0.0001
	α − pinene	5.14 / 0.006	0.48 / 0.62	4.41 / <0.0001
	β − caryophyllene	9.36 / <.0001	0.13 / 0.88	7.00 / <0.0001
	β − pinene	0.09 / 0.91	0.91 / 0.40	0.67 / 0.50

Results are shown for individual compounds per species averaged over male and female beetles, combined for genders and gender-dose interaction, and on compound-solvent comparisons, averaged over doses and male and female beetles

Data have been collected from 62 independent antennae of *C. populi* (35 females, 27 males) and 26 independent antennae of *P. vitellinae* (15 females, 11 males)

**Fig. 1:**
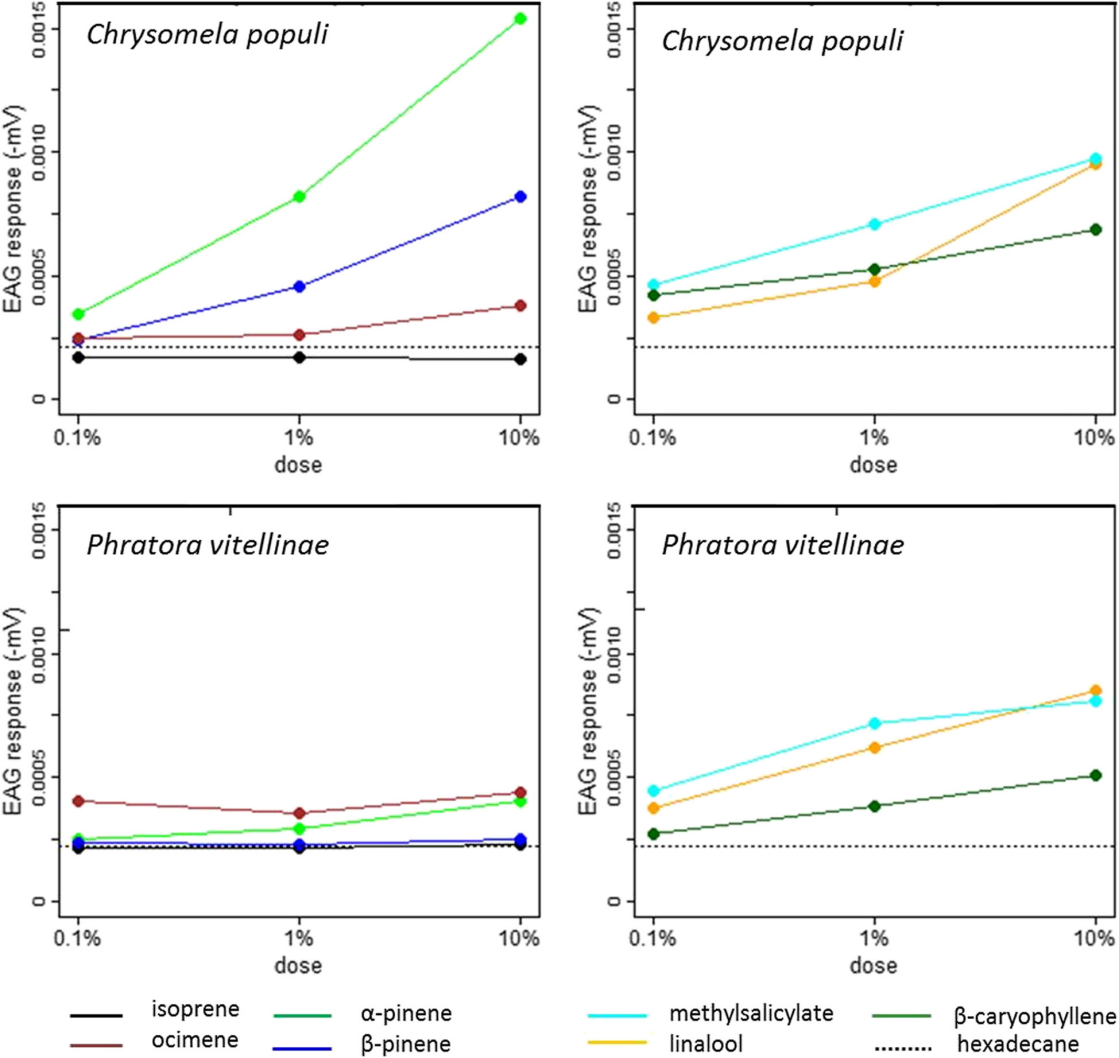
Dose–response curves for EAG responses of *Chrysomela populi* and *Phratora vitellinae* to different volatile compounds. The curves are based on the Mixed Linear Model. Dose is expressed as dilution (*v*/*v*) of the compound in hexadecane. EAG response (expressed as maximum amplitude of depolarization) is expressed in – mV. The mean absolute EAG response amplitude to hexadecane is shown by the *dashed line*

For *C. populi* significant dose–response relationships (in order of strength according to the F-statistic) were found for α-pinene, β-pinene, linalool, methylsalicylate, β-caryophyllene, and ocimene (Table 2; Fig. 1). For isoprene no significant dose–response relationship was found (*P* = 0.92). For *P. vitellinae* significant dose–response relationships were found for linalool, β-caryophyllene, methylsalicylate and α-pinene. No significant dose–response relationships were detected for ocimene, isoprene, and β-pinene (Fig. 1). Statistical comparison with the response to the solvent hexadecane showed that for *C. populi* the EAG amplitude in response to isoprene was significantly lower than the response to the solvent, which was not the case for *P. vitellinae*. With the exception of the β-pinene / solvent comparison in *P. vitellinae*, all other responses to test compounds were higher than to solvent (all *P* < 0.0001).

The results of the random part of the model are detailed in Additional file 7, 8: Table S5 and 9: Fig S3.

### VOC emission profiles differed between infested and non-infested IE and NE poplars

**Fig. 2:**
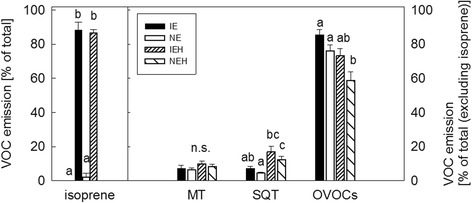
Volatile emission of isoprene emitting (IE) and non-emitting (NE) poplars with or without herbivory (H). Isoprene emission is presented as percentage of total emission, monoterpene (MT), sesquiterpene (SQT) and other VOC emissions are presented as percentages of total MT, SQT and OVOC emission (mean ± SE, *n* = 6 ± 1). Different letters indicate significant differences within each compound class between the genotype and herbivore treatment, *P* < 0.05. The individual compounds can be found in Additional file 3: Table S2 and the statistical analysis for the whole data in Additional file 8: Table S5

The foliage of infested poplar trees emitted several individual compounds and total sesquiterpenes more intensively than foliage from non-infested plants [Fig. 2; Additional files 4: Table S3, 8: Table S5]. (*E*,*E*)-α-farnesene was the most abundant sesquiterpene, with approximately two-fold higher emission rates from IE than NE plants. Alfa-cubebene, (*E*)-caryophyllene and germacrene D were other sesquiterpenes that together accounted for the relatively high total sesquiterpene emission rates from IE plants compared to NE plants.

Expressed as percentages the total monoterpene emission rates did not differ between the plants (Fig. 2, Additional file 8: Table S5). However, some individual monoterpenes (allo-ocimene, tricyclene) were significantly enhanced or tend to be enhanced ((*E*)-β-ocimene) only in infested IE genotypes [Additional files 4: Table S3, 8: Table S5]. In accordance, the absolute monoterpene emission rate of the IE plants, only, increased significantly after herbivory (Additional files 4: Table S3, 8: Table S5).

In addition to these poplar genotype-specific differences to herbivory, several other VOCs showed increased emissions after the onset of leaf feeding even if the percental emission rate of the other VOCs decreased (Fig. 2, Additional file 4: Table S3, 8: Table S5). From these compounds the increase of 2-ethylfuran and (*E*)-2-hexen-1-ol was more pronounced in IE plants than in the NE plants after infestation.

The emission of the homoterpene (*E*)-4, 8-dimethyl-1,3,7-nonatriene (DMNT) was, moreover, constitutively higher from the foliage of IE than from NE genotypes [Additional files 4: Table S3, 8: Table S5]. This observation is interesting because this homoterpene is known as a general herbivore-inducible compound [44, 45].

### *C. populi* larvae and adults showed no clear preference for NE or IE poplar leaves in Y-tube olfactometer or greenhouse bioassays

**Fig. 3:**
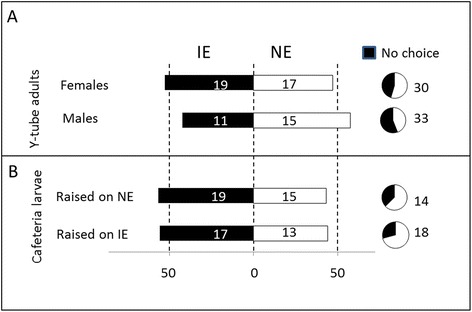
Behavioral responses of *Chrysomela populi* to isoprene emitting (IE) and non-emitting (NE) poplar. Response of *C. populi* adults (**a**) to volatiles released by IE and NE plants in a Y-tube olfactometer and response of larvae (**b**) in feeding choice-experiments. Bars represent the overall percentages of beetles choosing either of the odor sources; numbers in bars are the total numbers of beetles choosing that odor source. The percentage of insects not choosing is shown in pie charts. Binomial tests showed no significances between the choices

Supporting the fact that *C. populi* cannot sense isoprene, the adult beetles showed no preference to the volatiles from non-infested IE versus NE trees (Fig. 3A, Additional file 8: Table S5). Similarly, when larvae of *C. populi* were given a free choice in a choice-test setup to feed either on IE or on NE leaves, they did not prefer either plant type in the beginning (first choice) of the experiment (Fig. 3b, Additional file 8: Table S5).

**Fig. 4:**
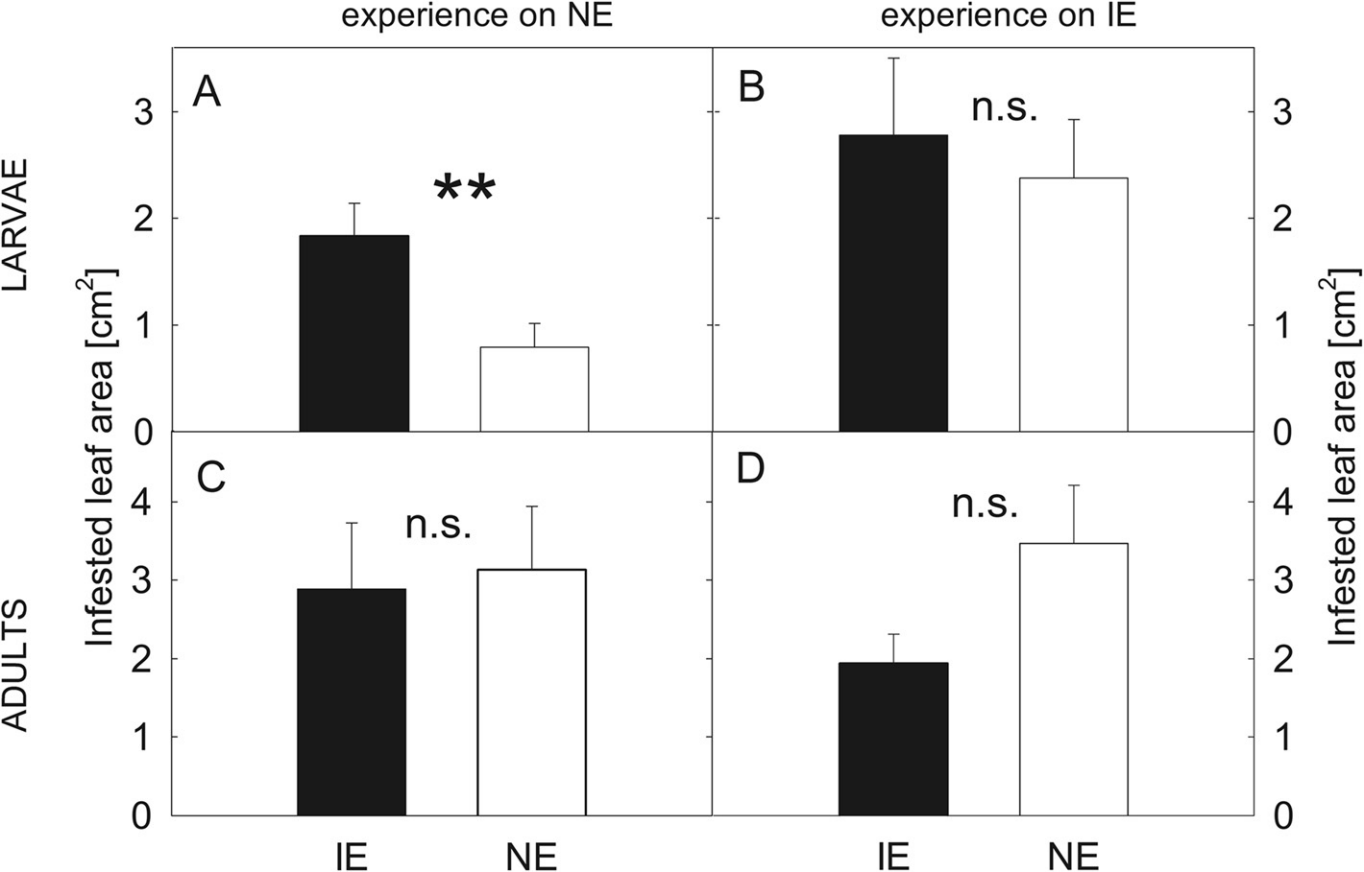
Feeding choice of *Chrysomela populi* on isoprene emitting and non-emitting poplar leaves. Feeding choice of 3^rd^ instar *C. populi* larvae (**a**, **b**) and adult beetles (**c**, **d**) on isoprene emitting (IE; ■) plants or non-emitting (NE; □) plants. The herbivores had previous feeding experience on NE (**a**, **c**) or IE plants (**b**, **d**) and they were offered a choice between IE and NE leaves. The infested leaf areas were analyzed from photographs by SigmaScan Pro5 computer program and are presented as mean ± SE, n (**a**) =35; (**b**) = 45; (**c**, **d**) = 30; asterisk indicates significant difference between the genotypes, *P* < 0.01 (detailed statistical analysis in Additional file 8: Table S5)

To perform the feeding choice-experiments, the hatched larvae were either raised on NE or IE plants until the 3^rd^ instar stage was reached. They were then given the choice between IE and NE leaves. We observed more feeding damage (Fig. 4a, Additional file 8: Table S5) on IE leaves when the larvae were reared on NE plants. By contrast, no difference in the feeding damage was detected when the larvae had previous experience on IE plants (Fig. 4b, Additional file 8: Table S5).

The choice experiments on leaves were also conducted with adult beetles, in whose no differences were found for theconsumption of the leaf material, independent of the previous experience (Fig. 4c, d, Additional file 8: Table S5).

**Fig. 5:**
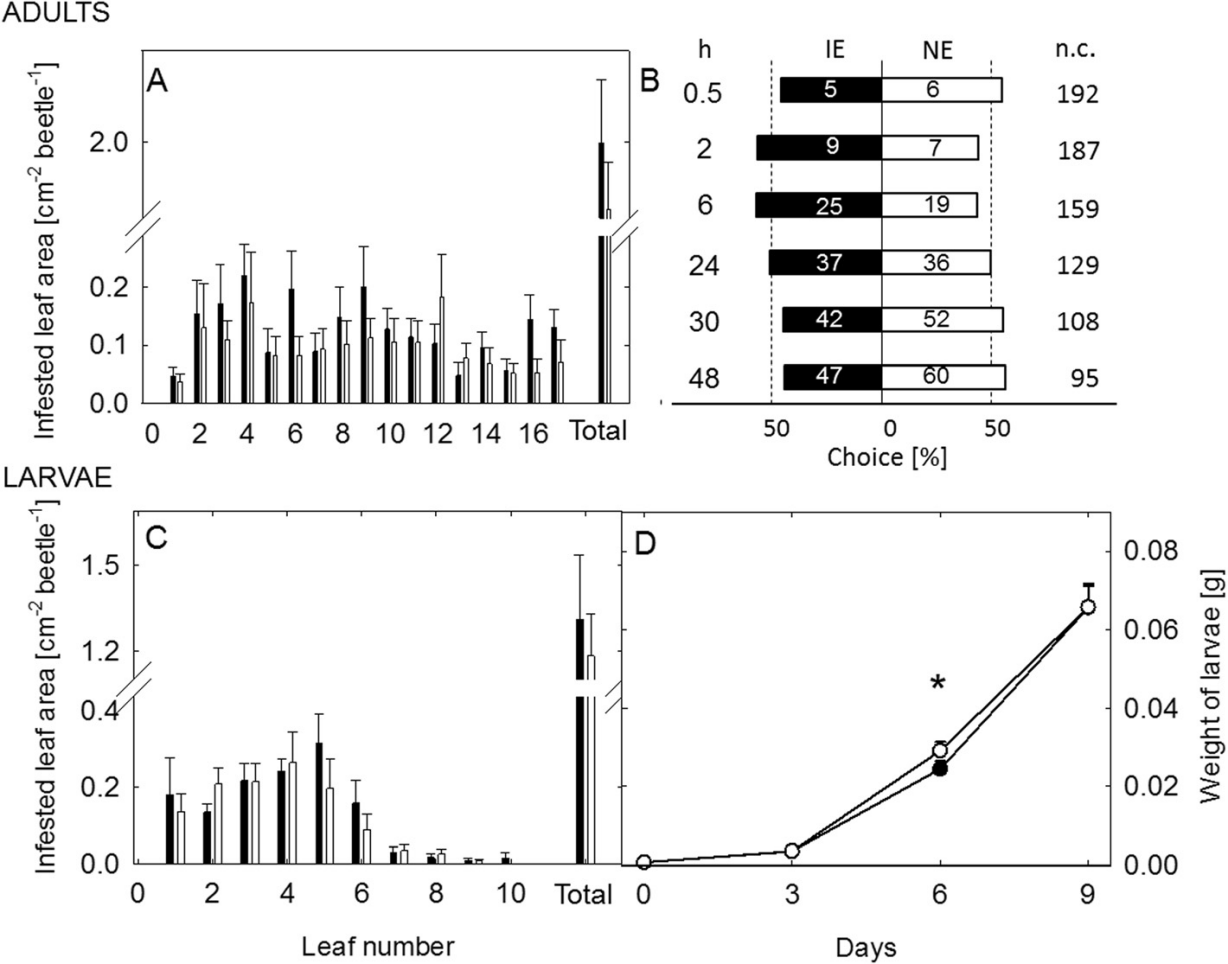
Feeding choice of *Chrysomela populi* on isoprene emitting and non-emitting poplar trees. Feeding choice of adult *C. populi* beetles and larvae on isoprene emitting (IE) and non-emitting (NE) plants in the greenhouse. The mean infested leaf area from apex until lowest leaf and in total by adult *C. populi* beetles (mean +/− SE, *n* =20) (**a**) and the choices of beetles (**b**) in a cage with two IE (■) and two NE (□) trees over 48 h-period. The beetle choices are shown for hours (h) 0, 5, 2, 6, 24, 30 and 48. Bars represent the overall percentages of beetles choosing either of the odor sources; numbers in bars are the total numbers of beetles choosing that odor source; n.c., no choice. The feeding choice of larvae offered either IE (■) or NE (□) trees over 10 days was assessed by the infested leaf area (individual leaves from apex until lowest leaf and total leaf area) (**c**) at the end of experiment and the development (**d**) of the larvae by weighing them every 3^rd^ day (mean ± SE; n: (**c**) = 8; (**d**) = 160 (beginning);121 ± 8 (3 days); 67 ± 1; (6 days); 33 ± 1 (10 days)). The infested leaf areas were analyzed from photographs by SigmaScan Pro5 computer program

No clear preference for any poplar genotype was observed when the adult beetles were offered a free choice to feed between whole IE and NE trees (*t* = 0.854, df = 38, *P* = 0,398, paired *t* test on total infested leaf area, Fig. 5a) Similarly no differences were found in choices of the adult beetles (Fig 5b, Additional file 8: Table S5). The larvae exhibited equal infestation rates (*t* = 0.483, df = 14, *P* = 0.636, *t* test on total infested leaf area) and reached in the end of the experiment similar body weights (Fig. 5d, Additional file 8: Table S5) on IE and on NE plants. On day six the larvae feeding on NE plants had slightly more body weight than the larvae feeding on IE plants (Fig 5d, Additional file 8: Table S5). No differences were found for the survival rate of the larvae on the genotypes (on average 25 % of the larvae reached the age of 10 days).

### *C. populi* preferred to feed on IE in field conditions

**Fig. 6:**
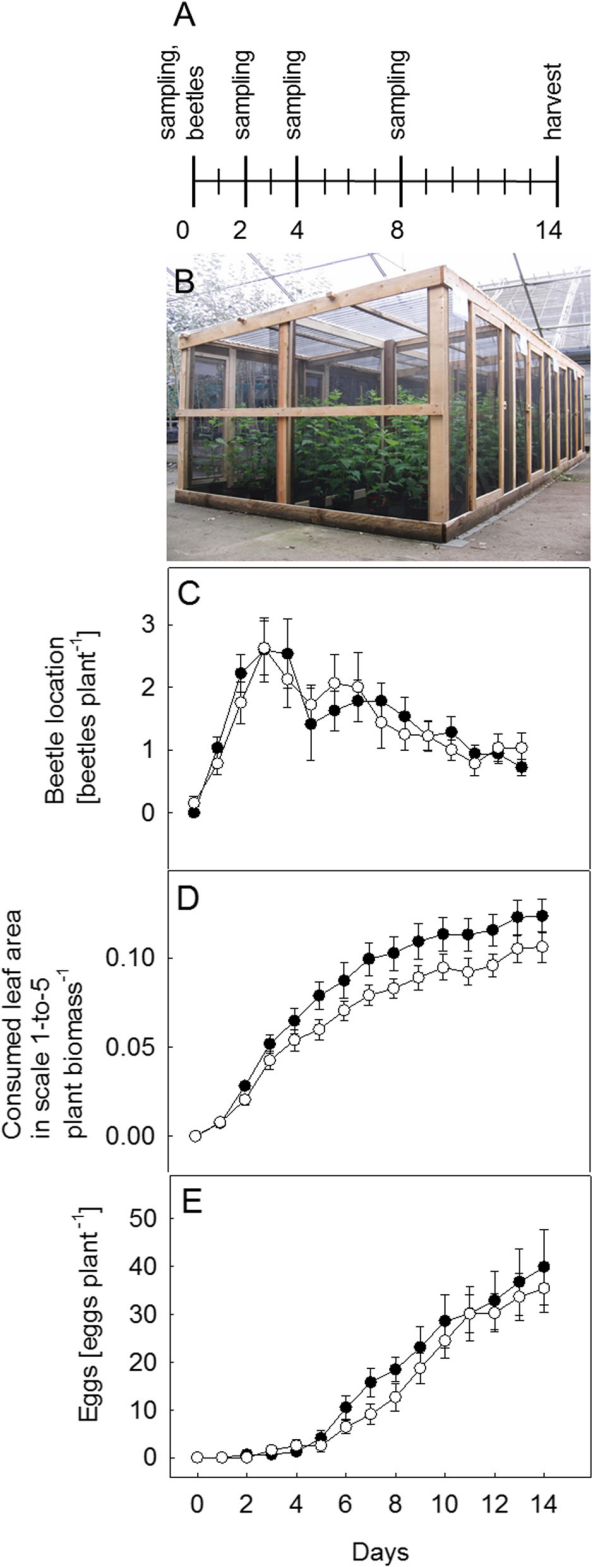
*C. populi* bioassay under field conditions. The experimental scheme shows the days of sampling, harvesting and the release of the beetles (**a**). A picture of the semi natural experimental cages (**b**) and *C. populi* location (**c**), leaf consumption (**d**) and laid eggs (**e**) on isoprene emitting (IE; ■) and non-emitting (NE; □) poplar trees in cages during a 14-day experiment. Consumed leaf area (**b**) is a visual approximate in which a scale 0-to-5 (0: no damage; 1 < 10 % infested leaf, 2: 20-to-25 %, 3: 25-to-50 %, 4: 50-to-75 % and 5 > 75 % infested leaf) was applied. Mean ± SE of 8 replicates is presented

No differences were found in the distribution of *C. populi* between the IE and NE genotypes under field conditions during a 14-day experiment in close-to natural conditions (experimental scheme and cages in Fig. 6a, b) (Fig. 6c, Additional file 8: Table S5). However, the beetles fed significantly more on the IE than on the NE trees over time (*P* < 0.01, Fig. 6d, Additional file 8). *C. populi* females tended to oviposit on IE compared to NE plants over time (*P* = 0.052, Fig. 6e, Additional file 8: Table S5). The magnitude of these differences, however, was small (10 ± 2 % less leaf area and 4 ± 15 % fewer eggs per plant).

The biomass of the trees did not show any differences between IE and NE or between the beetle-infested and non-infested trees at the end of the experiment [Additional file 8: Table S5, 10: Fig S4].

### Metabolome-wide changes depended on leaf development, herbivory and isoprene emission capacity

**Fig. 7:**
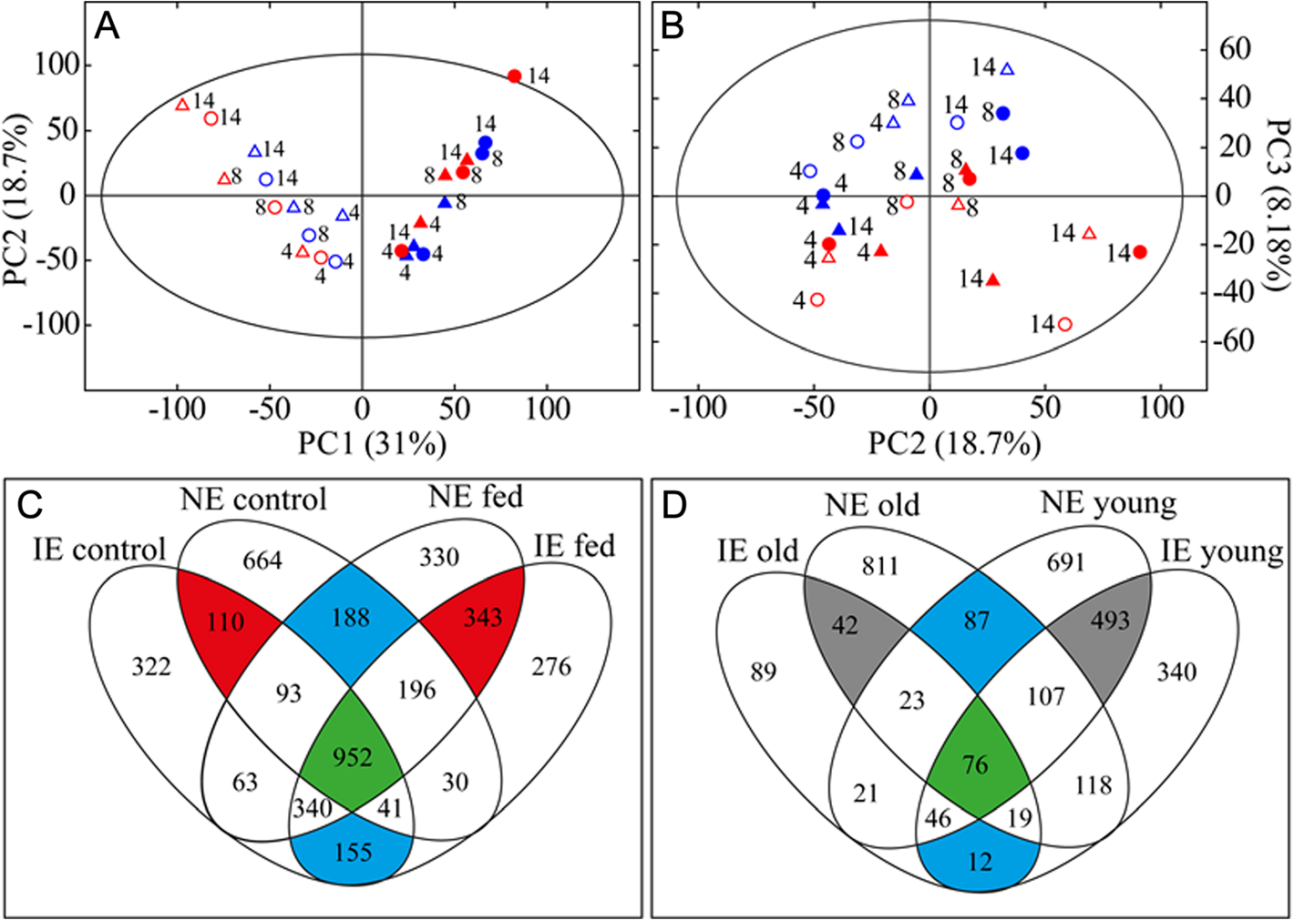
Metabolomics of *Chrysomela populi* infested isoprene emitting (IE) and non-emitting (NE) poplars under field conditions. Score scatter plots of Principal Component Analysis (PCA) of FT-ICR/MS metabolomics data showing (**a**) PC1 versus PC2 and (**b**) PC2 versus PC3. Symbols represent the poplar genotypes and leaf age (IE young leaf [○], NE young leaf [Δ], IE old leaf [●], NE old leaf [▲]). Red symbols represent infested and blue control plants. Venn-Diagram of discriminant masses (**c**) responsible for the separation of leaf developmental stage and (**d**) responsible for the separation of infested from control plants

PCA analysis revealed a leaf-age and time-point dependent clustering in the principle component 1 (PC1) × PC2 score plot (Fig. 7a). Metabolic differences between infested and control leaves were found in the score plot of PC2 × PC3. (Fig. 7b). Overall, the PCA model described leaf-age, beetle feeding and sampling time and explained 58 % of the total data variance. The PCA, however, did not show a clear separation between IE and NE plants.

To extract masses responsible for the observed PCA separation and to clarify whether NE and IE poplars responded differently to herbivory, separate OPLS-DA models for IE and NE poplars were calculated.

We compared the discriminant masses for the separation of leaf-age (control and infested plants) in IE and NE found by OPLS-DA in Venn-Plots (Fig. 7c). We observed a large proportion of leaf-age dependent discriminant masses that differ between NE (852 masses) and IE (477 masses), proving the existence of a metabolic genotype effect. Compared to the pronounced leaf-age and herbivore dependent metabolic differences, genotype dependent metabolic changes seemed to be of minor importance in this experimental setup.

**Fig. 8:**
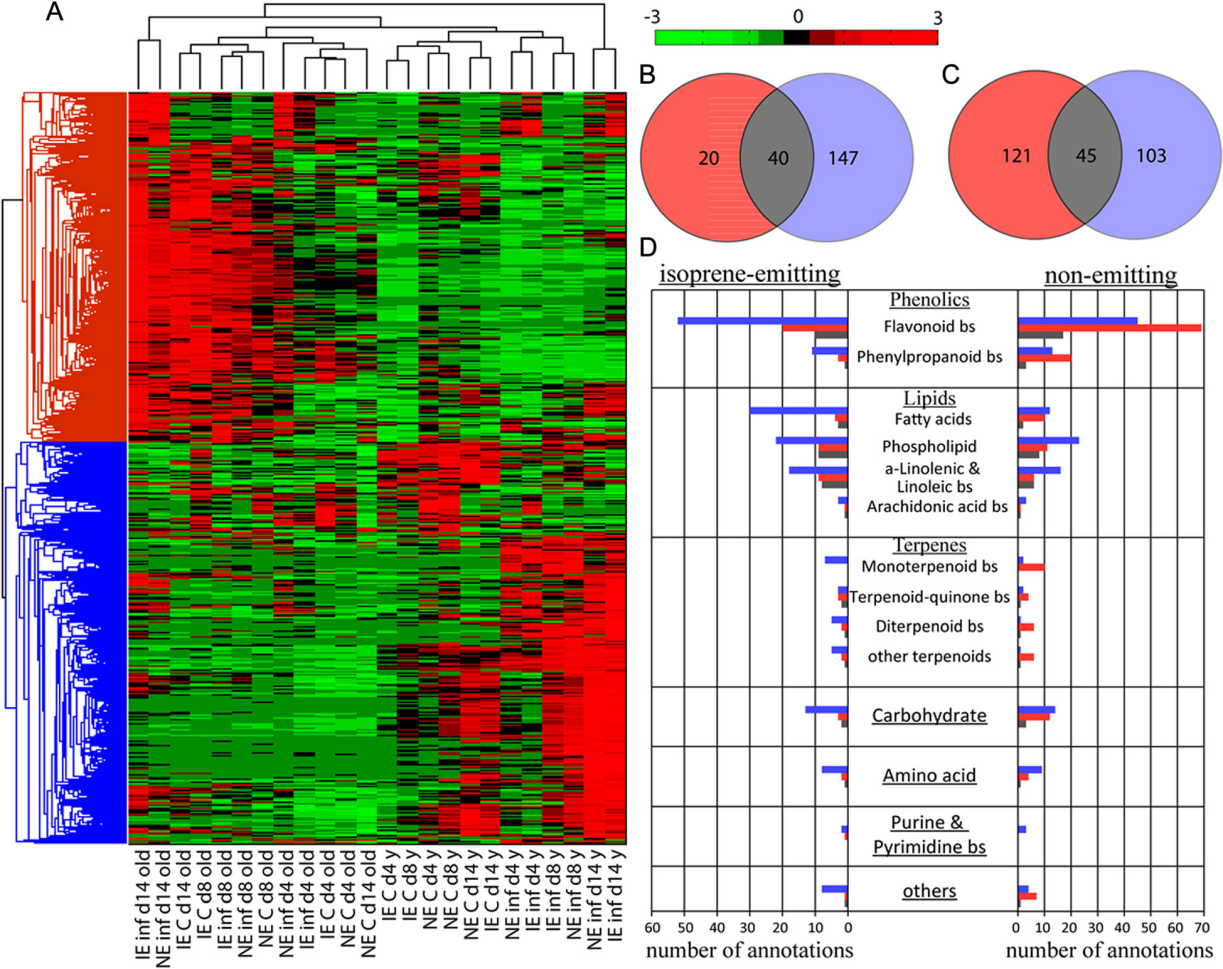
Genotype-, time- and herbivore feeding-dependent metabolic changes both in the infested and adjacent undamaged leaves. Comparison of the metabolomics between infested (young) and adjacent undamaged (old) leaves in isoprene emitting (IE) and non-emitting (NE) poplar on the days 4, 8 and 14 after the release of *Chrysomela populi*. Hierachical clustering analysis of FT-ICR/MS intensities of all discriminant masses (**a**). The Venn plot shows the annotated discriminant metabolites of infested young and old leaves in IE (**b**) and NE plants (**c**). The metabolite annotations are shown in the bar chart ordered according to compound classes, pathways and poplar genotypes (**d**). Young leaf: ■, old leaf: ■ and both leaves: ■, bs: biosynthesis

Next we investigated whether herbivory caused different metabolic adjustments in IE and NE poplars (Fig. 7d), which might explain the herbivore preference towards IE. Once again we found genotype dependent metabolic deviations in local (young) and adjacent undamaged (old) leaves (Fig. 7d). We detected, moreover, a more pronounced response in local and adjacent undamaged leaves in NE (811 adjacent undamaged and 691 local masses) than in the IE genotypes (89 adjacent undamaged and 340 local masses). Though, we also found a large portion of discriminant masses overlapping, which points out a conserved metabolic response to herbivory in local (493 masses) as well as in adjacent undamaged (42 masses) leaves. Additionally, 76 masses were discriminant for herbivory in both leaf types for IE and NE. Those metabolites were unsaturated fatty acids (FA), oxo-FA, phosphoglycerolipids, jasmonates and flavonoid glucosides. The 2956 masses discriminant for herbivory (Fig. 7d) were subjected to hierarchical clustering analysis (HCA) using the Pearson correlation coefficient (Fig. 8a). We found that young leaves clustered separately from old leaves and also subclustered in control and infested leaves.

Using databases, we were able to annotate 361 masses (12 %) as metabolites. To further elucidate the poplar’s response to herbivory, the metabolite changes were examined in young and old leaves under herbivory in more detail. Twenty and 147 metabolites showed significant changes after herbivory in old and young IE and NE leaves, respectively, whereas 121 and 103 metabolites changed in old and young NE leaves, respectively. In total, 40 metabolites changed in both young and old IE leaves and 45 compounds in young and old NE leaves (Fig. 8b-c). The metabolites could be grouped according to their respective compound classes and biosynthetic routes (Fig. 8d). This clustering revealed an influence of herbivory in both leaf age classes over various pathways. Overall, the data showed differential regulation of the phenolics metabolism: in NE poplars, more phenylpropanoids were abandoned in old leaves than in young leaves, whereas the opposite was observed for the IE poplars. Flavonoids, on the other hand, were upregulated in both the leaf ages in infested plants.

Furthermore, 3-dehydroquinate and shikimate, important precursors of plant phenolics, were upregulated in infested young leaves while no change was observed in adjacent undamaged leaves. Infested young leaves showed, moreover, up-regulation of phospholipids and in metabolites of the α-linolenic, linoleic and arachidonic acid biosynthetic pathways. A few of these metabolites, particularly those of the α-linolenic acid biosynthetic pathways, were also found to be discriminant in old leaves. In addition to these changes, we observed changes in the purine and pyrimidine metabolism in young leaves.

## Discussion

### *C. populi* or *P. vitellinae* cannot detect isoprene but respond to several other volatiles

An ecologically highly important result is that the leaf-feeding beetles *C. populi* and *P. vitellinae,* which are naturally present on high isoprene-emitting species, do not exhibit electroantennographic sensitivity to isoprene over a wide dosage range. It cannot be entirely excluded that very few antennal olfactory neurons are sensitive to isoprene and have been overseen by EAG recordings; however, isoprene itself is unlikely to act as an orientation cue. Any behavioural effects were likely caused by the indirect effects of the lack of isoprene production on leaf metabolism. Both beetle species were able to detect higher terpenoids, which may be used in beetle orientation. Young, developing poplar leaves have been previously shown to emit monoterpenes and sesquiterpenes rather than isoprene, whereas in older leaves isoprene is almost the only volatile terpenoid [21, 26, 46]. In agreement with the bioassay studies of [21], the present results show that *C. populi* can detect many terpenes emitted by young poplar leaves. Among the tested compounds α-pinene and β-pinene elicited the strongest responses in *C. populi.* These compounds accounted for 11 and 39 % of the monoterpene emission of *P*. x *canescens*, respectively, and are thus good candidate kairomones to *C. populi*. Ocimene, methylsalicylate, and β-caryophyllene, were also detected by this herbivore and may guide it to *P*. x *canescens*. In contrast, although *C. populi* can recognize linalool, linalool emission was not detected from *P. x. canescens*. In previous studies linalool accounted approximately for 20 % of the total monoterpene emission of *P. nigra* [21] and was also detected from the poplar hybrid *Populus tremula* x *tremuloides* [35], and thus, may play a role in *C. populi* interactions with these *Populus* spp.

Although the poplar specialists *C. populi* or *P. vitellinae* were not able to detect isoprene, previous studies on transgenic Arabidopsis and tobacco plants modified to emit isoprene have shown that other insect species such as the moth *M. sexta* or the parasitic wasp *D. semiclausum* can recognise this compound [15, 16]. The ecological meaning of this observation remains unclear e.g. because the host species parasitised by *D. semiclausum* is not known to feed on isoprene emitting plants. It is possible that isoprene, which is ubiquitously present in the canopy of poplar trees, is not a specific enough signal for *C. populi* or *P. vitellinae*.

In a previous field study, however, *P. vitellinae* preferred to stay on NE rather than IE plants [6]. The lack of isoprene emission cannot directly cause the preference of *P. vitellinae* for isoprene non-emitters, because the compound can physiologically not be detected by the herbivore. Metabolomic effects [6, 23, 24, 27] or other environmental interactions, such as the detected concurrent infection by pathogenic fungi [6], may have resulted in altered herbivore behaviour of *P. vitellinae* in [6].

### *C. populi* shows a slight preference to isoprene emitting plants

The repression of isoprene biosynthesis and emission in *P*. x *canescens* has direct and indirect effects on the plant’s physiology. The metabolic flux through the 2-C-methyl-D-erythritol-4-phosphate (MEP)-pathway was strongly reduced (99 %) in NE poplars as a consequence of the feedback inhibition of 1-deoxy-D-xylulose-5-phosphate synthase activity by the accumulation of plastidic dimethylallyl diphosphate [47]. The MEP pathway is required for monoterpene production, and its suppression may result in the diminished biosynthesis of monoterpenes. This suggestion is supported here by the reduced, herbivore-induced monoterpene emission rates. In addition, the emission of some individual terpene and non-terpene compounds was differentially induced in the infested IE and NE plants. As a consequence, less effective induction of herbivore-inducible VOCs may have occurred in NE plants and influenced the beetle responses in the choice experiments. Metabolic differences between the genotypes, moreover, might have influenced the beetles feeding choices (for discussion about metabolomics differences, please see the next section).

In the greenhouse bioassays, however, no clear preference for either of the genotypes was found. Some tendencies to IE plants were recorded in experiments with individual insects, but no preferences were found in the group experiments. In group experiments it is possible that herbivore behaviour differs from that of individual beetle, due to e.g. cues emitted by conspecifics [18]. In nature, however, several environmental factors, in addition to the conspecifics, influence plant physiology and herbivore feeding. In general, analysis under natural or at least semi-natural conditions is crucial to evaluate the ecological consequences of transgenic plants. Previous studies have shown that *Bacillus thuringiensis* (*Bt)* expressing poplar is better off than wild type in laboratory conditions but not in semi-natural conditions [48]. Our bioassays under field conditions revealed slightly less feeding on NE than on higher terpene-emitting IE trees despite the similar beetle abundances on the plants. Oviposition was also slightly reduced on the NE genotype, indicating moderate effects on poplar leaf beetle ecology. The detected slight differences in feeding did not affect the growth performance of the different genotypes. Previous studies showed that only a high herbivore pressure leads to differences in the biomass production of wild type and *Bt* poplar [29]. In the present study 3.75 herbivores per plant were introduced on the poplars, and the beetle number decreased during the experiment. Thus, it might be possible that under higher herbivore pressure as well the plant’s physiology as the herbivore feeding would be more affected. In general, in nature several factors, such as conspecifics and other animals, climate, microbes and other environmental factors can affect both, the beetle and poplar performance [18, 23] leading to different ecological consequences.

### Leaf age, genotype and herbivore feeding dependent differences in IE and NE poplar metabolomics under field conditions

Genetic modification may result in unintended effects on innate traits, such as it is shown for *Bt* poplars, in which among others content of the phenolic compounds was altered [29]. In the leaves of NE poplars, metabolome-wide shifts have been detected under different environmental conditions [6, 24, 27], most likely as the result of the adjustment of metabolic fluxes and transcriptional regulation and the need to replace isoprene functionality by other metabolites. The present results highlight metabolic changes in the defence chemistry in the NE compared to the IE genotypes. These changes, especially those observed in the biosynthesis of phenolic compounds, may have changed the leaf attractiveness for herbivores and could be responsible for the slight infestation preference of *C. populi* observed in our studies. It has been shown that alterations in foliar phenolic composition resulting from exposure of *Populus trichocarpa* to UV-B radiaton influenced leaf consumption from *Chrysomela scripta* (cottonwood leaf beetle) [49]. Furthermore, Boeckler *et al*. [50] showed that poplars genetically modified in tannin biosynthesis, results in an enhanced tannin content, which causes an increased palatability of leaves for herbivores due to a subsequent down-regulation of phenolic glycosides in this genotype. Compatible with these observations, we found that beetle-infested NE and IE addressed their phenolic metabolism, as well as their lipid metabolism, differently. These metabolomic alterations may reduce the attractiveness of NE leaves leading to the preference of *C. populi* to IE.

Nevertheless, the metabolome-wide changes due to leaf age, plant age, and herbivore feeding were much more pronounced than those induced by the transgenic modification. Several of the identified fatty acids can have a function in plant signalling. For example, α-Linolenic acid (18:3), a direct precursor of the signalling molecule jasmonic acid (JA) [51], was upregulated in infested leaves. A signalling molecule that may elicit stress and signalling networks [52], arachidonic acid and the salicylic acid-related plant signalling compound azelaic acid [53] were differentially regulated between young and old leaves. Such metabolic differences between leaf age classes may partially, among different emission profiles, cause the beetle preference to feed on young leaves [21]. Indeed, compared to the metabolomic differences between the infested young and adjacent undamaged old leaves, the genotype-dependent differences were minor.

## Conclusions

In this study, we demonstrated that naturally occurring leaf beetles (*C. populi* and *P. vitellinae*) on poplar are unable to sense isoprene. Nevertheless, minor differences in insect feeding choices were found, which may have been related to the changes in leaf volatile emission and metabolite composition in the NE genotype. Although *C. populi* beetles showed moderate preference to isoprene emitters, infestation did not affect overall plant biomass production. The present results and those of Behnke *et al*., [6] suggest that isoprene emission itself is of marginal importance for the susceptibility of poplar plantations to their natural herbivore *C. populi*. However, further studies in nature under different environmental conditions are instrumental to evaluate the NE poplar interaction with *C. populi*. It is evident that the lacking isoprene emission capacity leads to multiple constitutive and induced metabolic rearrangements in these genotypes that may be pronounced e.g. under additional abiotic or biotic stresses. Long-term and multi-factorial experiments in the field are necessary to comprehensively evaluate the ecological consequences of isoprene non-emitting poplars.

The changes in tree physiology may, moreover, have several, so far undetected, consequences for other various interactions of poplar plants with their environment. Especially interactions in which multiple partners are involved are difficult to predict. Isoprene *per se*, furthermore, can interfere with the communication between some insects and plants [15, 16] and might function e.g. as a repellent for species that are not searching for isoprene-emitters [15]. The role of isoprene in the tritrophic interactions (poplar-herbivore-parasitoid/predator) of a natural isoprene emitting species is yet to be elucidated.

## Additional files

Additional file 1:
**Experimental material for the bioassays.** The isoprene emitting (IE) and non-emitting (NE) poplar genotypes and the developmental stage and raring material of *Chrysomela populi* used in the insect bioassays. Detailed information for the poplar genotypes can be found in [5, 26]. Additional file 2:
**A schema of the olfactometer system.** Each cuvette was flushed with a flow of 1 L synthetic air mixed with 270 ppb CO_2_ (1). A flow of 500 ml was directed to each olfactometer arm (2), or alternatively, a flow of 100 mL was directed to the cartridges filled with adsorbent (3). The Y-tube had an internal diameter of 2,6 cm, two shorter arms (4, length 13,5 cm) and a longer arm (5, length 16,5 cm) in which the insects were introduced. Additional file 3:
**Characteristics of the volatile organic compounds (VOCs) presented in Additional file 2.** Characteristics of the VOCs sampled from isoprene emitting (IE) and non-emitting (NE), and *Chrysomela populi* infested or un-infested *P*. x *canescens* trees. CAS: Chemical Abstracts Service; RT: retention time; *I*: retention index calculated according to van Den Dool and Kratz [36]; m/z: mass to charge ratio. Additional file 4:
**Volatile organic compounds (VOCs) emitted by wild type and transgenic, infested and uninfested poplar trees.** VOCs emitted by isoprene emitting (IE) and non-emitting (NE), and *Chrysomela populi* infested or un-infested *P*. x *canescens* trees, mean ± SE, *n* = 6. The characteristics of the individual VOCs are presented in the Additional file 1. Different letters indicate statistically significant differences between genotypes and/or herbivore treatments, *P* < 0.05. Additional file 5:
**A scheme of an acclimatized cuvette for conducting bioassays.** The numbers indicate the inlet (1) and outlet (2) airflow. Additional file 6:
**Design of the field experiment.** For each treatment there were four plots, in which four individuals of two isoprene non-emitting genotypes (RA1, RA22) and two isoprene emitting genotypes (GUS, WT) were placed. In half of the plots (herbivore1-4) 60 *Chrysomela populi* individuals were added in the beginning of the experiment. Additional file 7:
**Statistical analysis and the results on the random part of the linear mixed model used for EAG data.**
Additional file 8:
**Statistical analyses.**
Additional file 9:
**Predicted relationships between stimulus order and EAG response by quadratic regression for individual antennae and overall.** Data from an individual antenna are shown as example. Additional file 10:
**The** ***Populus*** **x** ***canescens*** **biomass in field conditions.** The biomass of isoprene emitting (IE) and isoprene non-emitting (NE) and *Chrysomela populi* infested (H) and non-infested *Populus* x *canescens* trees in field conditions. The biomass of the aboveground (A) and belowground (B) parts was measured after 14-d lasting experiment.

## Abbreviations

*Bt*: *Bacillus thuringiensis*
VOC: Volatile organic compound
EAG: Electroantennography
FT-ICR/MS: Fourier transform ion cyclotron resonance mass spectrometer
GC-MS: Gas chromatograph-mass spectrometer
GFP: Green fluorescence protein
GLM: Generalized linear model
GLV: Green leaf volatile
GUS: β-glucuronidase
IE: Isoprene emitting
JA: Jasmonic acid
MEP: 2-C-methyl-D-erythritol-4-phosphate
NE: Isoprene non emitting
OPLS-DA: Orthogonal partial least squares discriminant analysis
ORN: Olfactory receptor neurons
OVOC: Other volatile organic compound
*Pc*ISPS: *Populus* x *canescens* isoprene synthase
PDMS: Polydimethylsiloxane
PCA: Principal component analysis
RNAi: RNA interference
SE: Standard error
SOA: Secondary organic aerosol
VIP: Sariable influence of projection score
