# Supplementary material for: Isoprene emission by poplar is not important for the feeding behaviour of poplar leaf beetles
Source: BMC Plant Biol. 2015 Jun 30;15:165. doi: 10.1186/s12870-015-0542-1 (PMC4486431; doi:10.1186/s12870-015-0542-1)
Supplement: Additional file 1: — Experimental material for the bioassays. The isoprene emitting (IE) and non-emitting (NE) poplar genotypes and the developmental stage and raring material of Chrysomela populi used in the insect bioassays. Detailed information for the poplar genotypes can be found in [5, 26]. [file 12870_2015_542_MOESM1_ESM.pdf]

Experiment	*Populus* x *canescens*						*Chrysomela populi*	
	IE			NE			Developmental stage	Rared on
	GUS2	GUS26	WT	RA1	RA2	RA22		
VOC analysis	x	x		x	x		Adults	IE
Olfactometer	x	x		x	x		Adults	IE
Choice studies (leaves, adult)	x	x		x	x		Adults	IE or NE
Choice studies (leaves, larvae)		x			x		3rd stage larvae	IE or NE
Choice studies (trees, adult)	x	x		x	x		Adults	IE
Choice studies (trees, larvae)	x	x		x	x		1st stage larvae	Remains of egg
Field experiment		x	x		x	x	Adults	Leaves from plantation
