# Supplementary figures and images for: Isoprene emission by poplar is not important for the feeding behaviour of poplar leaf beetles
Source: BMC Plant Biol. 2015 Jun 30;15:165. doi: 10.1186/s12870-015-0542-1 (PMC4486431; doi:10.1186/s12870-015-0542-1)

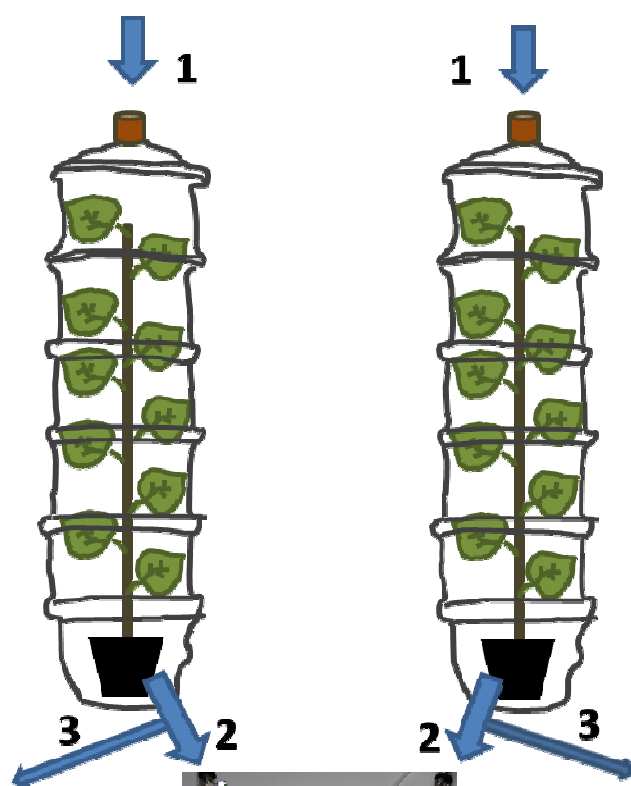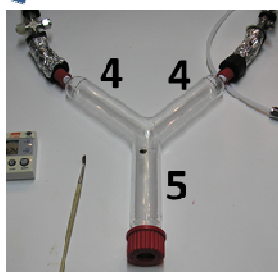

Supplement: Additional file 2: — A schema of the olfactometer system. Each cuvette was flushed with a flow of 1 L synthetic air mixed with 270 ppb CO2 (1). A flow of 500 ml was directed to each olfactometer arm (2), or alternatively, a flow of 100 mL was directed to the cartridges filled with adsorbent (3). The Y-tube had an internal diameter of 2,6 cm, two shorter arms (4, length 13,5 cm) and a longer arm (5, length 16,5 cm) in which the insects were introduced. [file 12870_2015_542_MOESM2_ESM.pdf]

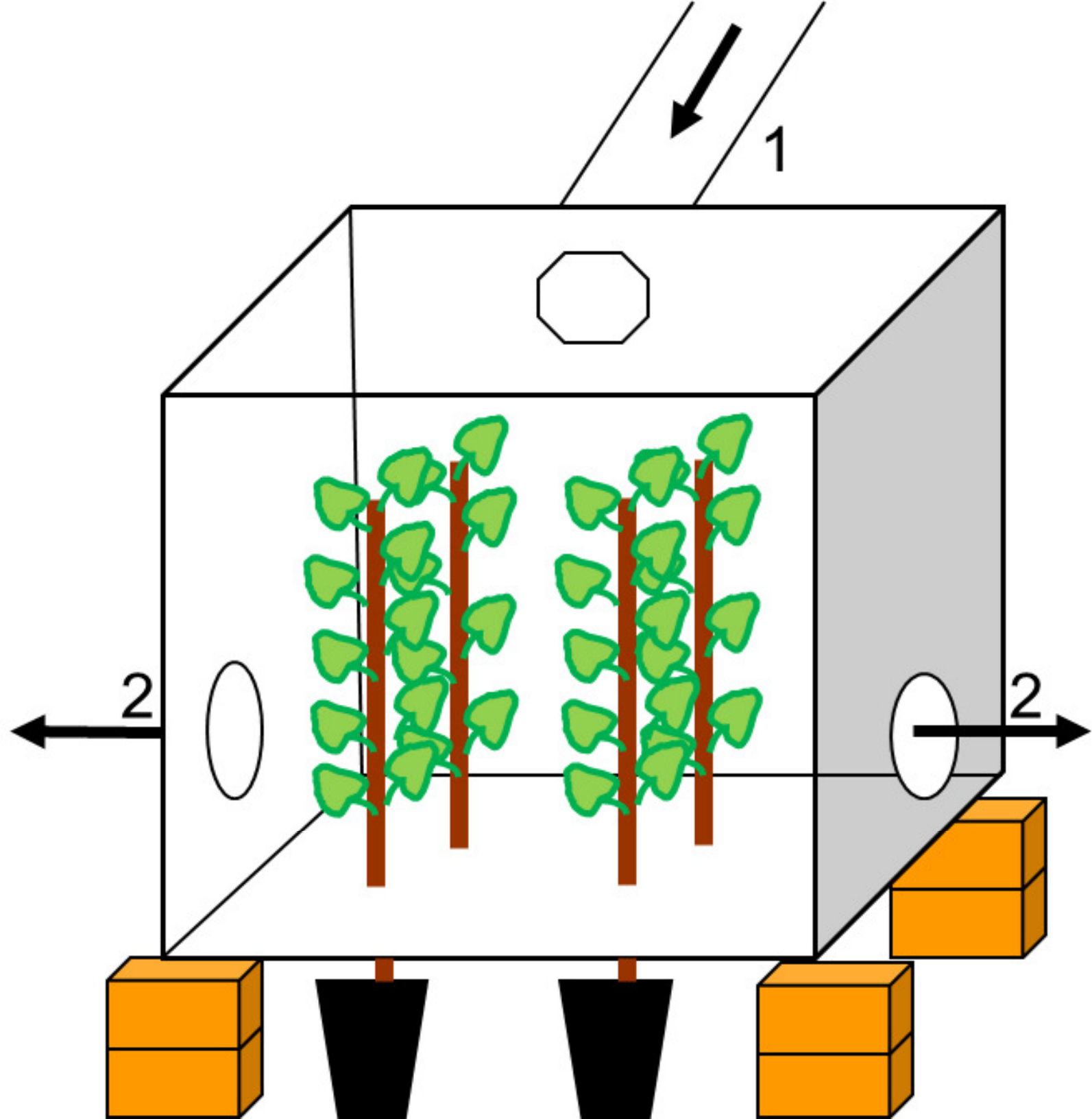

Supplement: Additional file 5: — A scheme of an acclimatized cuvette for conducting bioassays. The numbers indicate the inlet (1) and outlet (2) airflow. [file 12870_2015_542_MOESM5_ESM.pdf]

Progress of antenna sensitivity over time

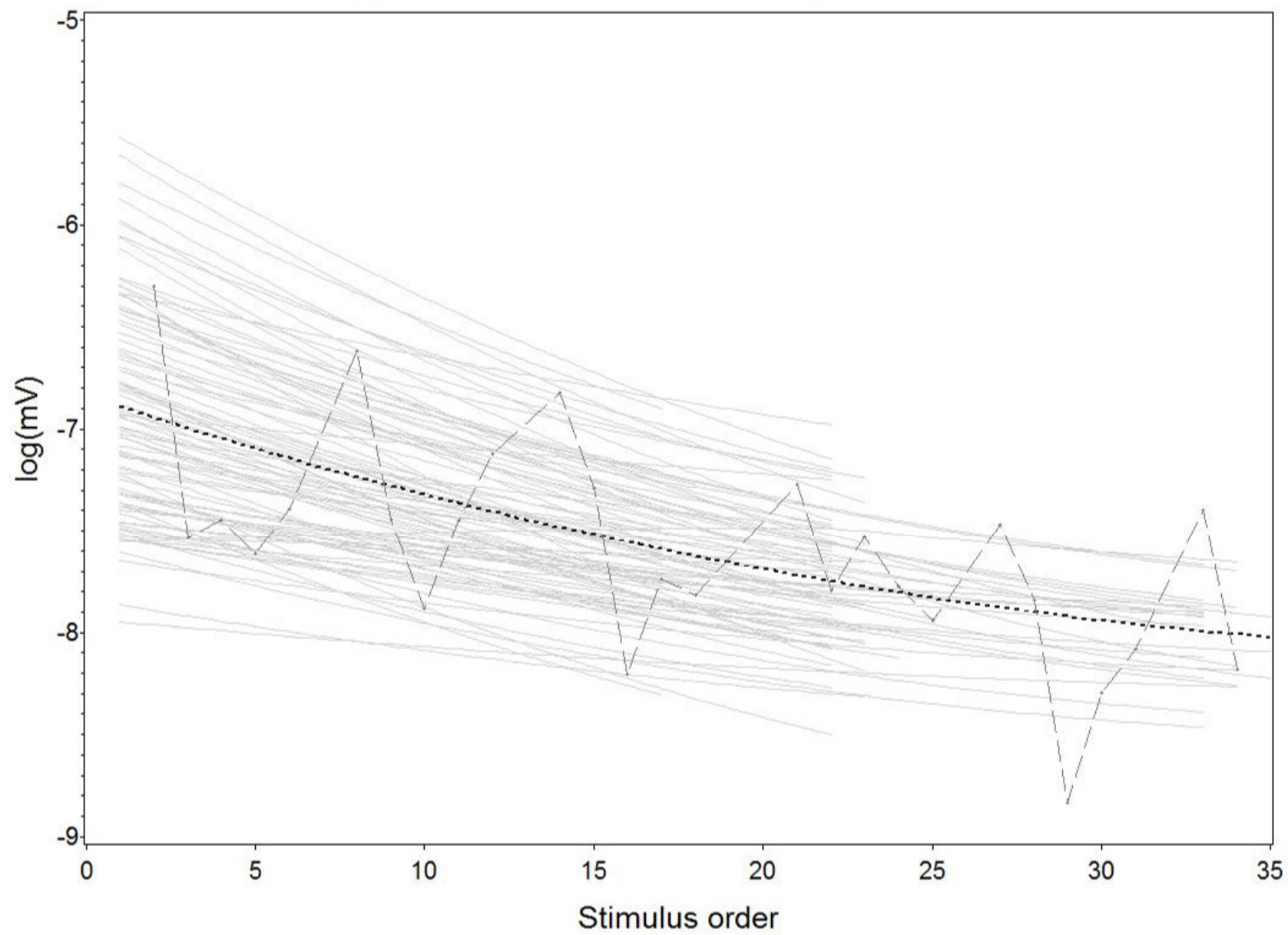

Supplement: Additional file 9: — Predicted relationships between stimulus order and EAG response by quadratic regression for individual antennae and overall. Data from an individual antenna are shown as example. [file 12870_2015_542_MOESM9_ESM.pdf]

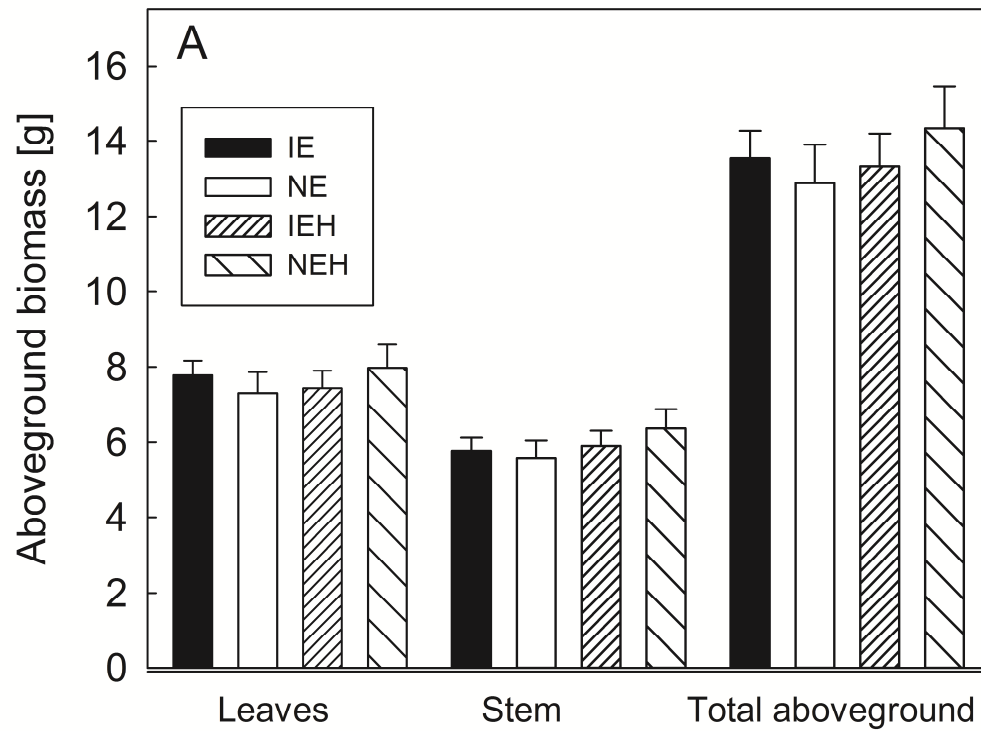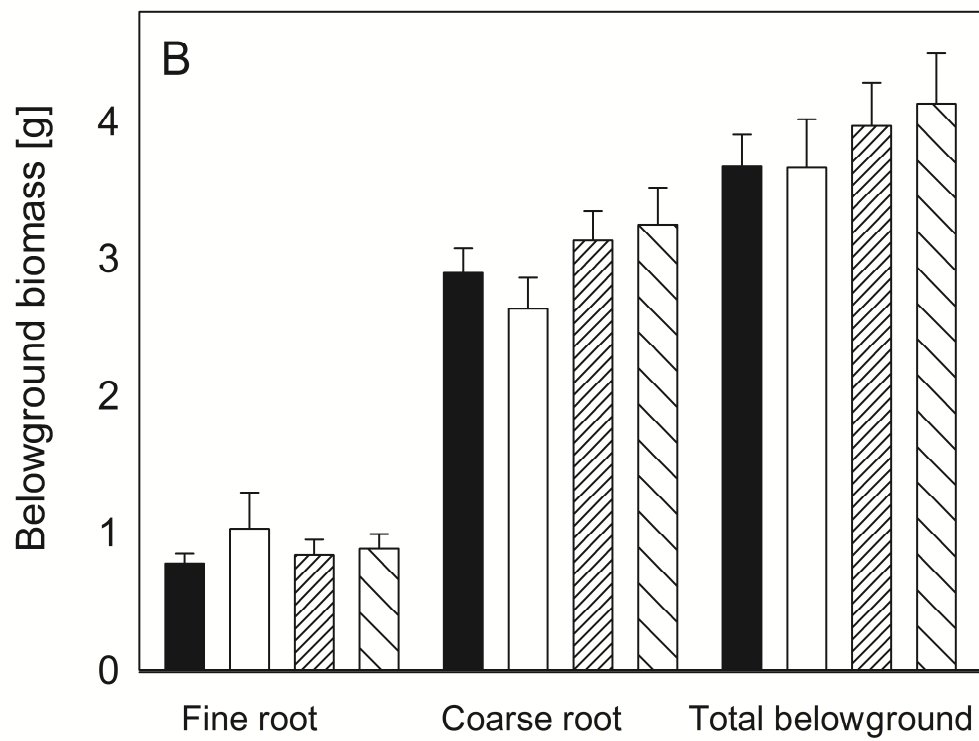

Supplement: Additional file 10: — The Populus x canescens biomass in field conditions. The biomass of isoprene emitting (IE) and isoprene non-emitting (NE) and Chrysomela populi infested (H) and non-infested Populus x canescens trees in field conditions. The biomass of the aboveground (A) and belowground (B) parts was measured after 14-d lasting experiment. [file 12870_2015_542_MOESM10_ESM.pdf]
