# Supplementary material for: Isoprene emission by poplar is not important for the feeding behaviour of poplar leaf beetles
Source: BMC Plant Biol. 2015 Jun 30;15:165. doi: 10.1186/s12870-015-0542-1 (PMC4486431; doi:10.1186/s12870-015-0542-1)
Supplement: Additional file 3: — Characteristics of the volatile organic compounds (VOCs) presented in Additional file 2. Characteristics of the VOCs sampled from isoprene emitting (IE) and non-emitting (NE), and Chrysomela populi infested or un-infested P. x canescens trees. CAS: Chemical Abstracts Service; RT: retention time; I: retention index calculated according to van Den Dool and Kratz [36]; m/z: mass to charge ratio. [file 12870_2015_542_MOESM3_ESM.pdf]

VOC	CAS registry no	RT	*I*	1st m/z	Absolute abundance (%) of 1st m/z
**Isoprene**	78795	5.99	578	67	25.1
**Monoterpenes**					
Tricyclene	508327	14.88	909	93	19.3
Cyclofenchene	488971	14.89	909	121	13.3
α -Thujene	2867052	14.96	911	77	24.7
α -Phellandrene	99832	15	912	91	11.0
α -Pinene	7785708	15.36	921	93	22.3
2- β -Pinene	127913	17.35	969	93	8.1
Myrcene	123353	17.62	975	93	6.9
Sabinene	3387415	19.73	1027	93	7.3
1,8-Cineole	470826	19.8	1028	71	27.1
Citronellal	106230	20.04	1034	71	17.8
(*E*)- β -ocimene	3779611	20.24	1039	93	19.5
γ -Terpinene	99854	20.88	1055	93	13.8
allo-Ocimene	673847	24	1131	79	4.8
Borneol	10385781	26.26	1185	95	6.2
β -Cyclocitral	432257	28.27	1234	81	8.7
**Monoterpenes total**					
**Sesquiterpenes**					
Bicyclogermacrene	24703353	33.06	1351	93	25.5
α -Ylangene	14912448	33.64	1365	161	17.8
α -Cubebene	17699148	33.64	1365	105	16.6
α -Copaene	3856255	34.88	1395	105	29.1
β -Elemene	515139	35.33	1406	93	12.9
Aromadendrene	489394	35.34	1406	161	26.6
(*Z*)-caryophyllene	13877935	36.07	1424	93	3.9
(*E*)-caryophyllene	87445	36.68	1439	93	2.6
β -Cubebene	13744155	37.03	1447	161	18.5
α -Guaiene	3691121	37.15	1450	105	25.3
Calarene	17334553	37.85	1467	105	7.5
α -Amorphene	23515880	37.87	1468	161	8.8
α -Humulene	6753986	38.05	1472	93	13.5
Germacrene-d	23986745	38.93	1493	161	6.2
α -Farnesene	502614	39.35	1504	93	17.2
d -Cadinene	483761	39.93	1518	161	4.9
Nerolidol	7212444	40.89	1541	69	18.7
Patchoulialcohol	5986550	43.48	1604	161	3.4
Sesquiterpenes total					
**Other BVOCs**					
propanenitrile, 2-methyl	78820	7.13	610	42	30.5
3-methylbutanal	590863	7.39	617	44	24.7
2-ethylfuran	3208160	8.11	637	81	15.1
(*E*)-1-Butyl-2-	38851706	10.26	797	55	28.2

methylcyclopropane					
(*Z*)-3-Hexen-1-ol	928961	12.09	841	67	4.2
(*E*)-2-Hexenal	505577	12.1	841	69	34.9
(*E*)-2-Hexen-1-ol	928950	12.43	849	82	3.9
1-Nonene	124118	13.41	873	55	2.1
2-Methyl-2-cyclopenten-1-one	1120736	14.02	888	67	10.8
(*E,E*)--2,4-Hexadienal	142836	14.26	894	81	3.5
Cumene	98828	14.87	909	105	10.5
(*Z*)-3-Hexen-1-ol acetate	3681718	18.28	992	67	5.1
p-Cymol	99876	19.11	1012	119	5.2
Salicylaldehyde	90028	20.37	1042	122	16.0
2-Methylphenol	95487	20.51	1046	108	5.7
Acetophenone	98862	21.29	1065	105	4.6
o-Isopropenyltoluene	7399497	22.39	1091	117	6.4
Methyl benzoate	93583	22.58	1096	105	16.9
(*E*)-4,8-dimethyl-1,3,7-nonatriene	0	23.32	1114	79	7.2
Benzeneethanol	60128	23.48	1118	91	4.2
Benzeneacetonitrile	140294	24.56	1144	117	5.9
(*Z*)-3-hexenyl iso-butyrate	41519237	26.51	1192	71	13.8
Methyl salicylate	119368	27.07	1205	120	9.6
1H-Indole	120729	31.35	1309	117	17.8
Eugenol	97530	33.71	1367	121	12.3
2-ethyl-naphthalene	939275	35.62	1413	141	6.5
Decyl acetate	112174	35.78	1417	55	11.6
β-Ionone	14901076	38.65	1487	177	8.0
dihydroactinidiolide	15356748	40.34	1528	11	24.3
(*Z*)-3-hexenyl benzoate	25152856	41.2	1549	105	27.6
n-Hexyl benzoate	6789884	41.36	1553	105	1.6
Unknown compound		46.83	1685	149	19.4
