# Supplementary material for: Isoprene emission by poplar is not important for the feeding behaviour of poplar leaf beetles
Source: BMC Plant Biol. 2015 Jun 30;15:165. doi: 10.1186/s12870-015-0542-1 (PMC4486431; doi:10.1186/s12870-015-0542-1)
Supplement: Additional file 4: — Volatile organic compounds (VOCs) emitted by wild type and transgenic, infested and uninfested poplar trees. VOCs emitted by isoprene emitting (IE) and non-emitting (NE), and Chrysomela populi infested or un-infested P. x canescens trees, mean ± SE, n = 6. The characteristics of the individual VOCs are presented in the Additional file 1. Different letters indicate statistically significant differences between genotypes and/or herbivore treatments, P < 0.05. [file 12870_2015_542_MOESM4_ESM.pdf]

VOCs [pmol m^{-2} s^{-1}] (in brackets % of total*****)				
Treatment	No herbivory		Herbivory	
P. x *canescens* genotypes	IE	NE	IE	NE
**Isoprene**	83.2±22.913^{A} (88.38)	0.38±0.38^{B}(2.14)	78.05±11.95^{A} (86.53)	/^{B}
**Monoterpenes**				
Tricyclene	/^{AB}	/^{A}	0.01±0.006^{B} (0.12)	0.01±0.004^{AB} (0.08)
Cyclofenchene	/	/	/	0.01±0.005 (0.09)
α –Thujene	/	0.02±0.015 (0.21)	0.01±0.005 (0.17)	0.04±0.011 (0.46)
α -Phellandrene	0.02±0.012 (0.27)	/	0.01±0.007 (0.14)	0.01±0.008 (0.12)
α –Pinene	0.06±0.041 (0.63)	0.02±0.016 (0.11)	0.11±0.059 (1.09)	0.11±0.062 (1.22)
2- β -Pinene	0.21±0.039 (3.28)	0.28±0.038 (4.03)	0.27±0.026 (2.89)	0.29±0.044 (3.40)
Myrcene	<0.01 (0.02)	0.02±0.012 (0.24)	0.02±0.007 (0.12)	0.01±0.050 (0.10)
Sabinene	/	<0.01 (0.02)	/	<0.01 (0.04)
1,8-Cineole	0.02±0.006 (0.21)	0.03±0.011 (0.37)	0.04±0.012 (0.62)	0.02±0.012 (0.18)
Citronellal	/	<0.01 (0.05)	<0.01 (0.01)	/
(*E*)- β -ocimene	0.15±0.041^{A} (2.28)	0.04±0.010^{B} (0.50)	0.48±0.151^{A} (4.18)	0.23±0.065^{A} (2.35)
γ -Terpinene	0.03±0.017 (0.42)	0.04±0.021 (0.70)	0.02±0.006 (0.29)	0.01±0.006 (0.11)
allo-Ocimene	<0.01^{A} (0.05)	/^{A}	0.02±0.005^{B} (0.15)	0.01±0.006^{AB} (0.07)
Borneol	<0.01 (0.01)	<0.01 (0.04)	0.01±0.003 (0.09)	0.01±0.004 (0.09)
β -Cyclocitral	/	/	<0.01 (0.01)	/
**Monoterpenes total**	0.49±0.136^{AB} (7.22)	0.46±0.100^{A} (6.29)	1.02±0.157^{B} (9.88)	0.75±0.182^{AB} (8.30)
**Sesquiterpenes**				
Bicyclogermacrene	/	/	<0.01 (0.02)	<0.01 (0.04)
α -Ylangene	0.01±0.008 (0.19)	/	/	0.01±0.007 (0.08)
α -Cubebene	<0.01^{A} (0.01)	/^{A}	0.01±0.005^{B} (0.09)	0.01±0.004^{B} (0.12)
α -Copaene	<0.01 (0.09)	0.01±0.001 (0.09)	0.01±0.001 (0.09)	0.01±0.002 (0.09)
β -Elemene	/	<0.01 (0.03)	0.01±0.006 (0.09)	0.01±0.006 (0.09)
Aromadendrene	/	0.01±0.003 (0.12)	/	/
(*Z*)-caryophyllene	<0.01 (0.02)	/	/	/
(*E*)-caryophyllene	0.09±0.023 (1.31)	0.09±0.038 (1.07)	0.11±0.029 (0.98)	0.07±0.013 (0.89)
β -Cubebene	0.01±0.004^{AB} (0.21)	0.01±0.003^{A} (0.07)	0.03±0.012^{AB} (0.22)	0.03±0.008^{B} (0.32)
α -Guaiene	<0.01 (0.12)	0.01±0.004 (0.15)	/ ()	/

Calarene	<0.01 (0.05)	<0.01 (0.02)	0.01±0.005 (0.07)	0.02±0.008 (0.23)
α-Amorphene	/	0.01±0.006 (0.17)	<0.01 (0.03)	/
α-Humulene	0.01±0.006 (0.21)	<0.01 (0.06)	0.02±0.007 (0.16)	0.01±0.004 (0.08)
Germacrene-d	0.09±0.028 (1.45)	0.07±0.013 (0.89)	0.2±0.078 (1.68)	0.2±0.061 (2.04)
α-Farnesene	0.24±0.050^{A} (3.33)	0.1±0.025^{B} (1.35)	1.51±0.449^{C} (13.19)	0.75±0.179^{C} (8.02)
d-Cadinene	0.01±0.001 (0.23)	0.02±0.007 (0.28)	0.02±0.006 (0.22)	0.03±0.011 (0.38)
Nerolidol	/	/	<0.01 (0.02)	/
Patchoulialcohol	<0.01 (0.02)	0.01±0.003 (0.10)	<0.01 (0.02)	<0.01 (0.01)
Sesquiterpenes total	0.49±0.078^{A} (7.24)	0.33±0.061^{A} (4.41)	1.95±0.545^{B} (16.87)	1.16±0.273^{B} (12.39)
**Other BVOCs**				
propanenitrile, 2-methyl	/	/	0.02±0.010 (0.16)	0.01±0.009 (0.15)
3-methylbutanal	/^{A}	/^{A}	0.02±0.004^{B} (0.19)	0.01±0.010^{AB} (0.14)
2-ethylfuran	/^{A}	0.04±0.027^{ABC} (0.31)	0.1±0.013^{B} (1.10)	0.04±0.017^{C} (0.46)
(*E*)-1-Butyl-2-methylcyclopropane	/	0.01±0.011 (0.07)	<0.01 (0.06)	<0.01 (0.05)
(*Z*)-3-Hexen-1-ol	1.10±0.256 (15.15)	1.08±0.28 (13.14)	2.1±0.739 (17.95)	1.05±0.307 (11.97)
(*E*)-2-Hexenal	/	/	0.15±0.155 (0.89)	0.55±0.444 (4.11)
(*E*)-2-Hexen-1-ol	/^{A}	/^{A}	0.11±0.037^{B} (0.83)	0.03±0.035^{AC} (0.17)
1-Nonene	/	/	<0.01 (0.07)	0.01±0.009 (0.19)
2-Methyl-2-cyclopenten-1-one	/	<0.01 (0.04)	0.01±0.003 (0.05)	<0.01 (0.03)
(*E,E*)--2,4-Hexadienal	/	/	0.02±0.010 (0.15)	<0.01 (0.03)
Cumene	0.02±0.007 (0.33)	0.01±0.009 (0.29)	/	0.01±0.003 (0.09)
(*Z*)-3-Hexen-1-ol acetate	3.87±0.936 (52.90)	4.52±1.042 (56.31)	3.52±1.012 (28.75)	2.41±0.718 (24.43)
p-Cymol	0.01±0.005 (0.19)	0.01±0.006 (0.07)	0.02±0.008 (0.17)	0.03±0.010 (0.32)
Salicylaldehyde	0.03±0.008^{A} (0.47)	0.05±0.010^{A} (0.75)	0.42±0.086^{B} (3.71)	0.58±0.317^{AB} (4.68)
2-Methylphenol	0.01±0.008 (0.09)	0.01±0.007 (0.11)	/	0.01±0.006 (0.08)
Acetophenone	/	/	0.04±0.024 (0.54)	0.06±0.03 (0.67)
o-Isopropenyltoluene	/	/	0.01±0.007 (0.07)	0.01±0.009 (0.15)
Methyl benzoate	0.02±0.014^{AB} (0.18)	0.01±0.005^{A} (0.04)	0.05±0.016^{B} (0.51)	0.05±0.015^{B} (0.57)
(*E*)-4,8-dimethyl-1,3,7-nonatriene	0.69±0.133^{A} (9.56)	0.15±0.018^{B} (2.16)	0.66±0.269^{A} (5.45)	0.26±0.059^{AB} (2.75)
Benzeneethanol	0.02±0.009^{A} (0.31)	0.03±0.004^{AB} (0.48)	0.10±0.025^{C} (0.81)	0.13±0.078^{BC} (1.11)
Benzeneacetonitrile	<0.01^{A} (0.03)	/^{A}	0.07±0.013^{B} (0.61)	0.12±0.041^{B} (1.17)
(*Z*)-3-hexenyl iso-butyrate	0.01±0.006 (0.09)	<0.01 (0.04)	0.03±0.011 (0.20)	0.03±0.012 (0.24)
Methyl salicylate	0.40±0.136 (5.10)	0.11±0.009 (1.70)	1.05±0.517 (9.23)	0.35±0.092 (3.65)

1H-Indole	0.01±0.004 (0.20)	<0.01 (0.03)	0.03±0.020 (0.23)	0.03±0.022 (0.22)
Eugenol	/^{A}	/^{A}	0.04±0.009^{B} (0.39)	0.06±0.033^{B} (0.58)
2-ethyl-naphthalene	<0.01 (0.03)	/	/	/
Decyl acetate	/	/	<0.01 (0.03)	/
β-Ionone	/	/	0.01±0.009 (0.09)	/
dihydroactinidiolide	/^{A}	/^{A}	0.01±0.003^{B} (0.10)	/^{A}
(*Z*)-3-hexenyl benzoate	0.08±0.041 (0.92)	0.06±0.029 (0.62)	0.07±0.012 (0.65)	0.04±0.009 (0.37)
n-Hexyl benzoate	/	/	0.01±0.007 (0.04)	<0.01 (0.01)
Unknown compound	/^{A}	/^{A}	0.02±0.005^{B} (0.21)	0.02±0.009^{AB} (0.24)
TOTAL other BVOCs	6.27±1.481 (85.54)	6.09±1.393 (76.16)	8.68±1.440 (73.25)	5.93±1.576 (58.61)

*for isoprene percentage of total emission; for others % of total emission excluding isoprene
