# Supplementary material for: Isoprene emission by poplar is not important for the feeding behaviour of poplar leaf beetles
Source: BMC Plant Biol. 2015 Jun 30;15:165. doi: 10.1186/s12870-015-0542-1 (PMC4486431; doi:10.1186/s12870-015-0542-1)
Supplement: Additional file 6: — Design of the field experiment. For each treatment there were four plots, in which four individuals of two isoprene non-emitting genotypes (RA1, RA22) and two isoprene emitting genotypes (GUS, WT) were placed. In half of the plots (herbivore1-4) 60 Chrysomela populi individuals were added in the beginning of the experiment. [file 12870_2015_542_MOESM6_ESM.pdf]

Control 1			
Wt<br>10	Ra 1<br>47	GUS 26<br>132	RA 22<br>83
RA 22<br>87	Wt<br>12	Ra 1<br>56	GUS 26<br>137
GUS 26<br>139	RA 22<br>100	Wt<br>17	Ra 1<br>66
Ra 1<br>67	GUS 26<br>141	RA 22<br>89	Wt<br>40

Herbivore 1			
Wt<br>4	Ra 1<br>42	GUS 26<br>121	RA 22<br>81
RA 22<br>104	Wt<br>11	Ra 1<br>48	GUS 26<br>125
GUS 26<br>131	RA 22<br>105	Wt<br>22	Ra 1<br>76
Ra 1<br>57	GUS 26<br>146	RA 22<br>120	Wt<br>31

Control 3			
RA 22<br>103	Wt<br>18	Ra 1<br>58	GUS 26<br>123
GUS 26<br>128	RA 22<br>109	Wt<br>26	Ra 1<br>64
Ra 1<br>68	GUS 26<br>130	RA 22<br>115	Wt<br>35
Wt<br>39	Ra 1<br>70	GUS 26<br>150	RA 22<br>118

Herbivore 3			
RA 22<br>91	Wt<br>7	Ra 1<br>53	GUS 26<br>140
GUS 26<br>143	RA 22<br>97	Wt<br>15	Ra 1<br>61
Ra 1<br>62	GUS 26<br>147	RA 22<br>98	Wt<br>20
Wt<br>28	Ra 1<br>71	GUS 26<br>153	RA 22<br>107

Control 2			
RA 22<br>119	GUS 26<br>160	Ra 1<br>78	Wt<br>19
Wt<br>14	RA 22<br>108	GUS 26<br>158	Ra 1<br>74
Ra 1<br>63	Wt<br>13	RA 22<br>106	GUS 26<br>151
GUS 26<br>144	Ra 1<br>60	Wt<br>9	RA 22<br>86

Herbivore 2			
RA 22<br>95	GUS 26<br>126	Ra 1<br>44	Wt<br>5
Wt<br>27	RA 22<br>113	GUS 26<br>152	Ra 1<br>73
Ra 1<br>72	Wt<br>25	GUS 26<br>111	RA 22<br>142
GUS 26<br>133	Ra 1<br>65	Wt<br>6	RA 22<br>102

Control 4			
Wt<br>29	RA 22<br>92	GUS 26<br>135	Ra 1<br>51
Ra 1<br>54	Wt<br>23	RA 22<br>93	GUS 26<br>136
GUS 26<br>154	Ra 1<br>69	Wt<br>8	RA 22<br>101
RA 22<br>117	GUS 26<br>155	Ra 1<br>79	Wt<br>34

Herbivore 4			
Wt<br>24	RA 22<br>96	GUS 26<br>129	Ra 1<br>49
Ra 1<br>59	Wt<br>32	RA 22<br>99	GUS 26<br>148
GUS 26<br>156	Ra 1<br>52	Wt<br>33	RA 22<br>110
RA 22<br>112	GUS 26<br>159	Ra 1<br>77	Wt<br>38
