# Supplementary material for: Isoprene emission by poplar is not important for the feeding behaviour of poplar leaf beetles
Source: BMC Plant Biol. 2015 Jun 30;15:165. doi: 10.1186/s12870-015-0542-1 (PMC4486431; doi:10.1186/s12870-015-0542-1)
Supplement: Additional file 7: — Statistical analysis and the results on the random part of the linear mixed model used for EAG data. [file 12870_2015_542_MOESM7_ESM.pdf]

Methods

Statistical analysis of EAG results

The fixed part of the model consists of main effects of insect species, gender, compound and compound concentration, and their interactions. Taking the analogy of a split-plot experiment, factors species and gender were defined as main plot factors associated with antennae, whereas compound and concentration were subplot factors, changing within antennae. Variables order and order^2 were used as covariates in both models to allow for an overall trend of EAG response over time. Instead of analyzing the original EAG recordings y (in –mV), we analysed $\ln(-y+0.0001)$ with the small constant added to avoid boundary problems near zero. The transformed variable resulted in a more symmetrical distribution of residuals.

As in [42], we have chosen the random parts of the mixed model to consist of two components: 1) random coefficients, allowing antennae to follow their own quadratic regression line over time; as proxy for time we took the order of the stimulus, defined as the [serial number *minus* 15], taking values in the range –14 to 20; the number of observations per antenna varied between 17 and 35; 2) random effects of days; EAG responses show slight variation among days beyond residual variation and variation already captured by the random coefficients part of the model; 3) residual error.

Results

EAG results; the random part of the model

The results on the random part of the model and covariates are presented in additional files 8 and 9. The random variation of the EAG response of *Chrysomela populi* and *Phratora vitellinae* was modelled to stem from three sources; estimated variance components are shown in an additional file [Additional file 8].

1) Random variation of quadratic regression lines per antenna over time. The variation due to the random coefficients is visualized in an additional file [Additional file 9] showing predicted regression lines per antenna. To illustrate the large variability between antennae, take the intercept variance of 0.104, which corresponds to standard deviation 0.32. Taking extreme antennae to differ 4 σ's, their difference in intercept is $4\times0.32 = 1.3$ units. Transforming to the original mV scale, the most sensitive antenna may show $e^{1.3} \approx 3.6$ times higher responses than the least sensitive antenna. Antennal sensitivity declined over time with a quadratic trend. Data from an individual and rather typical antenna has been plotted along. The overall estimated quadratic regression line for covariate order is shown in black, with highly significant linear (-0.019) and quadratic (0.00058) coefficients (Table 1).

2) Between-day variability. The day-to-day variance is relatively low: 0.019, corresponding to standard deviation $\approx$ 0.14, so that we may see differences up to 0.5 unit (or a 1.7-fold multiplication factor on the original mV scale) due to days.

3) Residual variability. The residual variance is estimated to be 0.099, roughly equal to the intercept variance of the antenna.
