# Supplementary material for: Isoprene emission by poplar is not important for the feeding behaviour of poplar leaf beetles
Source: BMC Plant Biol. 2015 Jun 30;15:165. doi: 10.1186/s12870-015-0542-1 (PMC4486431; doi:10.1186/s12870-015-0542-1)
Supplement: Additional file 8: — Statistical analyses. [file 12870_2015_542_MOESM8_ESM.pdf]

Results for hypothesis tests of species and gender effects on control compounds in EAG data.

Compound	Species main (F-val / P-val) (df=1,78)	Gender main (F-val / P-val) (df=1,78)	Species × gender interaction (F-val / P-val) (df=1,78)
(*Z*)-3-hexenyl-acetate	26.83 / <.0001	1.02 / 0.32	1.48 / 0.23
hexadecane	0.060 / 0.81	0.24 / 0.63	0.51 / 0.48

**Estimated variances for the different sources of variation of the random part of the Mixed Linear Model applied on EAG data (Additional file 7).**	
Source of variation	Estimated variance
Intercept	0.104
linear coefficient	0.00011
quadratic coefficient	1.56e-7
Day	0.019
Residual	0.099

Statistical analysis of total VOC emission (percentages, Figure 2)*				
	df	Kruskall-Wallis *P*		Mann-Whitney *P*
isoprene	3	0.000	IE-IEH	0.465
			IE-NE	0.004
			IE-NEH	0.003
			IEH-NE	0.002
			IEH-NEH	0.002
			NE-NEH	0.317
monoterpenes	3	0.255		
sesquiterpenes	3	0.005	IE-IEH	0.088
			IE-NE	0.068
			IE-NEH	0.045
			IEH-NE	0.010
			IEH-NEH	0.391
			NE-NEH	0.004
other VOCs	3	0.015	IE-IEH	0.088
			IE-NE	0.100
			IE-NEH	0.004
			IEH-NE	0.775
			IEH-NEH	0.086
			NE-NEH	0.025

*isoprene emitting: IE; isoprene non-emitting: NE; herbivory: H

**Statistical analysis of individual VOC compounds and compound groups (Additional file 4)***				
	df	Kruskall-Wallis		Mann-Whitney
		P		*P*
Isoprene	3	0.000	IE-IEH	0.808
			IE-NE	0.004
			IE-NEH	0.003
			IEH-NE	0.002
			IEH-NEH	0.002
			NE-NEH	0.317
Tricyclene	3	0.049	IE-IEH	0.053
			IE-NE	1.000
			IE-NEH	0.082
			IEH-NE	0.036
			IEH-NEH	0.707
			NE-NEH	0.059
Cyclofenchene	3	0.100		
α –Thujene	3	0.054		
α -Phellandrene	3	0.328		
α –Pinene	3	0.403		
2- β -Pinene	3	0.358		
Myrcene	3	0.398		
Sabinene	3	0.716		
1,8-Cineole	3	0.498		
Citronellal	3	0.611		
(*E*)- β -ocimene	3	0.002	IE-IEH	0.062
			IE-NE	0.011
			IE-NEH	0.410
			IEH-NE	

			IEH-NEH	,004
			NE-NEH	,199
				,004
γ -Terpinene	3	0.381		
allo-Ocimene	3	0.019	IE-IEH	0.035
			IE-NE	0.273
			IE-NEH	0.486
			IEH-NE	0.005
			IEH-NEH	0.186
			NE-NEH	0.140
Borneol	3	0.099		
β -Cyclocitral	3	0.167		
Monoterpenes total	3	0.022	IE-IEH	0.420
			IE-NE	1.000
			IE-NEH	0.201
			IEH-NE	0.010
			IEH-NEH	0.116
			NE-NEH	0.550
Bicyclogermacrene	3	0.611		
α -Ylangene	3	0.184		
α -Cubebene	3	0.009	IE-IEH	0.046
			IE-NE	0.273
			IE-NEH	0.022
			IEH-NE	0.015
			IEH-NEH	0.886
			NE-NEH	0.007
α -Copaene	3	0.123		

β -Elemene	3	0.198		
Aromadendrene	3	0.020	IE-IEH	1.000
			IE-NE	0.082
			IE-NEH	1.000
			IEH-NE	0.042
			IEH-NEH	1.000
			NE-NEH	0.059
(*Z*)-caryophyllene	3	0.284		
(*E*)-caryophyllene	3	0.794		
β -Cubebene	3	0.038	IE-IEH	0.193
			IE-NE	0.224
			IE-NEH	0.100
			IEH-NE	0.070
			IEH-NEH	0.775
			NE-NEH	0.006
α -Guaiene	3	0.063		
Calarene	3	0.056		
α-Amorphene	3	0.063		
α-Humulene	3	0.270		
Germacrene-d	3	0.062		
α-Farnesene	3	0.000	IE-IEH	0.004
			IE-NE	0.045
			IE-NEH	0.006
			IEH-NE	0.003
			IEH-NEH	0.153
			NE-NEH	0.004
d-Cadinene	3	0.354		

Nerolidol	3	0.047	IE-IEH	0.110
			IE-NE	1.000
			IE-NEH	1.000
			IEH-NE	0.082
			IEH-NEH	0.082
			NE-NEH	1.000
Patchoulialcohol	3	0.799		
Sesquiterpenes total	3	0,001	IE-IEH	0.007
			IE-NE	0.273
			IE-NEH	0.006
			IEH-NE	0.003
			IEH-NEH	0.391
			NE-NEH	0.004
propanenitrile, 2-methyl	3	0.151		
3-methylbutanal	3	0.004	IE-IEH	0.009
			IE-NE	1.000
			IE-NEH	0.361
			IEH-NE	0.005
			IEH-NEH	0.084
			NE-NEH	0.317
2-ethylfuran	3	0.007	IE-IEH	0.003
			IE-NE	0.176
			IE-NEH	0.034
			IEH-NE	0.082
			IEH-NEH	0.010
			NE-NEH	0.732
(*E*)-1-Butyl-2-methylcyclopropane	3	0.817		

(*Z*)-3-Hexen-1-ol	3	0.706		
(*E*)-2-Hexenal	3	0.091		
(*E*)-2-Hexen-1-ol	3	0.003	IE-IEH	0.009
			IE-NE	1.000
			IE-NEH	0.361
			IEH-NE	0.005
			IEH-NEH	0.043
			NE-NEH	0.317
1-Nonene	3	0.253		
2-Methyl-2-cyclopenten-1-one	3	0.254		
(*E,E*)--2,4-Hexadienal	3	0.031	IE-IEH	0.053
			IE-NE	1.000
			IE-NEH	0.361
			IEH-NE	0.036
			IEH-NEH	0.087
			NE-NEH	0.317
Cumene	3	0.162		
(*Z*)-3-Hexen-1-ol acetate	3	0.668		
p-Cymol	3	0.367		
Salicylaldehyde	3	0.004	IE-IEH	0.004
			IE-NE	0.144
			IE-NEH	0.068
			IEH-NE	0.003
			IEH-NEH	0.568
			NE-NEH	0.055
2-Methylphenol	3	0.705		
Acetophenone	3	0.092		

o-Isopropenyltoluene	3	0.285		
Methyl benzoate	3	0.010	IE-IEH	0.086
			IE-NE	0.416
			IE-NEH	0.065
			IEH-NE	0.005
			IEH-NEH	0.775
			NE-NEH	0.008
(*E*)-4,8-dimethyl-1,3,7-nonatriene	3	0.01	IE-IEH	0.372
			IE-NE	0.006
			IE-NEH	0.028
			IEH-NE	0.015
			IEH-NEH	0.668
			NE-NEH	0.055
Benzeneethanol	3	0.021	IE-IEH	0.027
			IE-NE	0.100
			IE-NEH	0.043
			IEH-NE	0.032
			IEH-NEH	0.721
			NE-NEH	0.055
Benzeneacetonitrile	3	0	IE-IEH	0.004
			IE-NE	0.273
			IE-NEH	0.005
			IEH-NE	0.002
			IEH-NEH	0.253
			NE-NEH	0.002
(*Z*)-3-hexenyl iso-butyrate	3	0.395		
Methyl salicylate	3	0.065		

1H-Indole	3	0.293		
Eugenol	3	0.000	IE-IEH	0.003
			IE-NE	1.000
			IE-NEH	0.004
			IEH-NE	0.002
			IEH-NEH	1.000
			NE-NEH	0.002
2-ethyl-naphthalene	3	0.284		
Decyl acetate	3	0.488		
β-Ionone	3	0.167		
dihydroactinidiolide	3	0.002	IE-IEH	0.024
			IE-NE	1.000
			IE-NEH	1.000
			IEH-NE	0.015
			IEH-NEH	0.015
			NE-NEH	1.000
(*Z*)-3-hexenyl benzoate	3	0.34		
n-Hexyl benzoate	3	0.621		
Unknown compound	3	0.026	IE-IEH	0.024
			IE-NE	1.000
			IE-NEH	0,082
			IEH-NE	0.015
			IEH-NEH	0.769
			NE-NEH	0.059
TOTAL other BVOCs	3	0.481	IE-IEH	0.223
			IE-NE	0.715
			IE-NEH	0.855

		IEH-NE	0.199
		IEH-NEH	0.253
		NE-NEH	0.749

*isoprene emitting: IE; isoprene non-emitting: NE; herbivory: H

**Statistical analysis of adult and larvae choices (Figures 3 and 5B)**			
	n	Z	*P* value (Binomial test)
Olfactometer (Fig. 3A)			
Female	36	-0.17	0.432
Male	26	-0.59	0.278
Choices of larvae (Fig. 3B)			
Raised on NE	34	-0.51	0.305
Raised on IE	30	-0.55	0.291
Choices of adults at hour (Fig. 5B)			
0.5h	11	0	0.500
2h	16	-0.25	0.401
6h	44	-0.75	0.226
24h	73	0	0.500
30h	94	-0.93	0.176
48h	107	-1.16	0.123

**Statistical analysis of adult and larvae feeding choices (Figure 4)**				
	n	df	*U*	*P* value (Wilcoxon signed- rank test)
Experience on NE				
Larvae	35	34	-2.380	0.017
Adult	30	29	-0.394	0.694
Experience on IE				
Larvae	45	44	-0.122	0.903
Adult	30	29	-1.389	0.165

Statistical analysis of the weight gain of larvae on whole trees (Figure 5D)*

	df1	df2	F	*P* value (ANOVA)
Days				
0	1	238	0.247	0.620
3	1	189	0.630	0.428
6	1	125	4.199	0.043
9	1	44	0.007	0.935

*For normally distributed data natural logarithm was calculated before analysis.

Statistical analysis on the beetle behavior in the field experiment (Figure 6).

	df1	Wald Chi-Squared	*P* value (General linear model)
Beetle location	1	0.139	0.709
Consumed leaf area	1	36.534	0.000
Eggs	1	3.772	0.052

Statistical analysis on poplar biomass in field conditions. (Additional file 9).

	df1	*t*	*P* value (*t* test)
leaves	62	0.740	0.462
stem	62	0.451	0.653
total aboweground	62	0.611	0.544
fine roots	62	0.822	0.414
coarse roots	62	0.963	0.339
total belowground	62	0.117	0.907
